# Encaging palladium(0) in layered double hydroxide: A sustainable catalyst for solvent-free and ligand-free Heck reaction in a ball mill

**DOI:** 10.3762/bjoc.13.160

**Published:** 2017-08-14

**Authors:** Wei Shi, Jingbo Yu, Zhijiang Jiang, Qiaoling Shao, Weike Su

**Affiliations:** 1 National Engineering Research Center for Process Development of Active Pharmaceutical Ingredients, Collaborative Innovation Center of Yangtze River Delta Region Green Pharmaceuticals, Zhejiang University of Technology, Hangzhou, 310014, Zhejiang, China

**Keywords:** ball milling, Heck reaction, layer double hydroxides, solvent-free, supported Pd catalyst

## Abstract

In this paper, the synthesis of a cheap, reusable and ligand-free Pd catalyst supported on MgAl layered double hydroxides (Pd/MgAl-LDHs) by co-precipitation and reduction methods is described. The catalyst was used in Heck reactions under high-speed ball milling (HSBM) conditions at room temperature. The effects of milling-ball size, milling-ball filling degree, reaction time, rotation speed and grinding auxiliary category, which would influence the yields of mechanochemical Heck reactions, were investigated in detail. The characterization results of XRD, ICP–MS and XPS suggest that Pd/MgAl-LDHs have excellent textural properties with zero-valence Pd on its layers. The reaction results indicate that the catalyst could be utilized in HSBM systems to afford a wide range of Heck coupling products in satisfactory yields. Furthermore, this catalyst could be easily recovered and reused for at least ﬁve times without signiﬁcant loss of catalytic activity.

## Introduction

High-speed ball milling (HSBM)-assisted transition metal-catalyzed cross-coupling reactions such as Heck, Suzuki, Sonogashira and Glaser reactions are still unusual methods for the formation of C–C bonds [1–7], but the method arouse considerable attention because of an environmentally benign and solvent-free synthesis approach as well as high efficiency and good atom economy, which is desirable in the fields of chemistry, materials science, biology, pharmaceutical, dyestuff, agriculture and so forth [8–12].

**Scheme 1:**
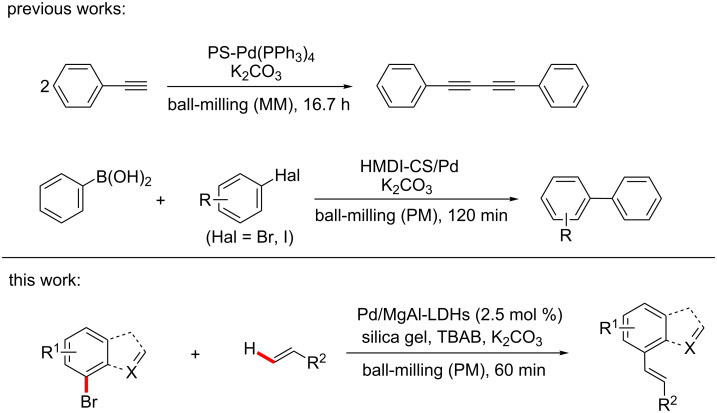
Supported catalysts in cross-coupling reactions. MM represents mixer mill; PM represents planetary mill.

The homogeneous palladium salts along with phosphine- or nitrogen-based ligands were employed as the traditional catalyst systems not only in solution-based C–C cross coupling [13–16] reactions but also in mechanically activated Heck [4,17–22], Suzuki [23–26], and Sonogashira [5,27–28] coupling reactions. The limitations of which are obviously unstable ligands and expensive Pd catalysts. Furthermore, the contamination of the coupled products with unacceptable Pd species led to a hard separation and recycling of homogeneous catalyst systems. In our previous study [4] we reported a ball-milling Heck reaction catalyzed by Pd(OAc)_2_. Although the catalyst showed the satisfactory reactivity, it was difficult to recover. Thus, Pd catalysts anchored on heterogeneous solid support materials such as MCM-41 [29], alumina [30], silica [31], carbon nanotubes [32], microporous polymers [33], SBA-15 [34], or some dendrimers [35] were preferred to develop a ligandless and recyclable catalyst system. However, to the best of our knowledge, only a few of supported Pd catalysts were used in mechanochemically assisted coupling reactions because of the low mechanical strength of the catalysts, the active component of which is easy to leach and deactivate under HSBM conditions. Mack and co-workers [36] reported a kind of polymer supported Pd(PPh_3_)_4_ catalyst with high activity in a Glaser reaction (Scheme 1). They found that the catalyst could only be recycled twice without the addition of the PPh_3_ ligand and the Pd component was significantly leached from polymer support after each run. Cravotto et al. [37] used an ultrasound-assisted method to prepare Pd catalysts immobilized on modified chitosan (Scheme 1). Although these catalysts were found to be effective in the Suzuki reaction after three cycles, the modiﬁcation conditions of chitosan were rigorous.

As catalyst-supported material, layered double hydroxides (LDHs) have received much attention in the organic catalysis for its excellent properties such as low costs, high specific surface area, double-layered structure, anion exchange capacity, high mechanical stability and chemical stability [38–43]. Our previous studies have proved that LDH catalysts could be successfully applied in the degradation of organic pollutants [44–45]. Bai and co-worker [46] synthesized Pd/SDS–LDHs by using an ultrasonic method, which exhibited excellent activity in Suzuki reactions. Jiang et al. [47] demonstrated that LDH-supported on alkaline materials performed higher catalytic activity in coupling reactions than that on acidic-supported materials. In the present work, co-precipitation was used for fabricating MgAl-LDHs with nitrate anions, followed by introducing disodium tetrachloropalladate (Na_2_PdCl_4_) into the LDH interlayer by the ion exchange method. The prepared hybrid LDHs were then reduced by hydrazine hydrate (N_2_H_4_·H_2_O) to obtain the Pd catalyst supported on MgAl-LDHs (Pd/MgAl-LDHs). The as-prepared Pd/MgAl-LDH catalyst was further applied in representative cross-coupling Heck reactions under HSBM conditions (Scheme 1) by using a planetary ball mill (Pulverisette 7, Fritsch, Germany). The inﬂuence of milling-ball filling degree (Φ_MB_), reaction time (*t*), milling-ball size (*d*_MB_) and rotation speed (*n*), along with catalyst loading, alkaline type and grinding auxiliary category were further investigated in detail.

## Results and Discussion

### Characteristics of prepared materials

**Figure 1:**
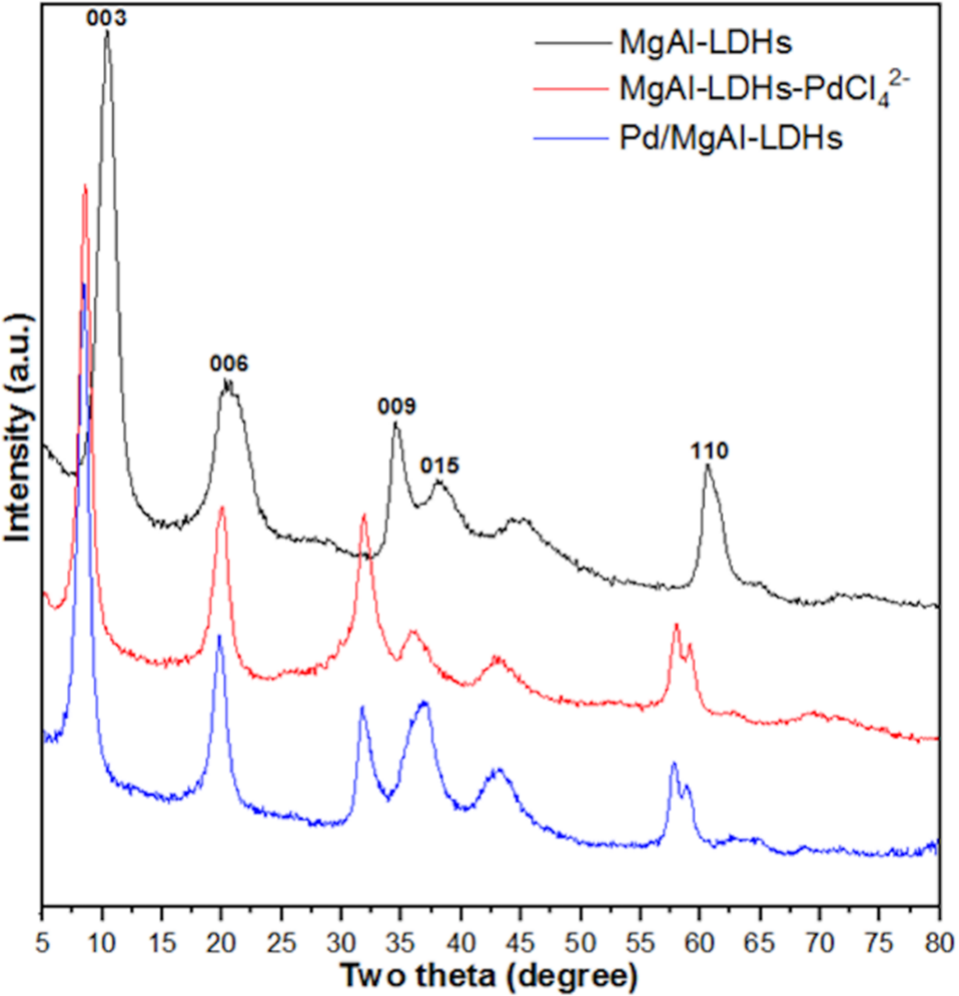
The XRD patterns for the samples of MgAl-LDHs, MgAl-LDHs-PdCl_4_^2−^ and Pd/MgAl-LDHs.

We initially prepared the Pd/MgAl-LDHs catalyst as described in the Experimental section (see Supporting Information File 1). Figure 1 shows the powder XRD patterns of MgAl-LDHs, MgAl-LDHs-PdCl_4_^2−^ and Pd/MgAl-LDHs at 2θ *=* 5–80°. All samples have diffraction peaks located around 10°, 20°, 33°, 38°, 60°, indexing to (003), (006), (009), (015), (110) reflections, which indicates the highly neat degree and well-crystallinity structure of LDH materials without phase impurities apparent. Moreover, the MgAl-LDHs presents an interlayer distance of 0.82 nm from the basal spacing of *d*_003_, which matches the results well for the intercalation of nitrate (NO_3_^−^) into MgAl-LDHs in literature [48]. In the MgAl-LDHs-PdCl_4_^2−^ sample, the (003) plane shifted to the lower position of 8.8°, resulting to an expansion of interlayer spacing of 1.01 nm from 0.82 nm. These phenomena suggest that PdCl_4_^2−^ successfully intercalated into the MgAl-LDHs interlayers. As compared with MgAl-LDHs and MgAl-LDHs-PdCl_4_^2−^, the catalyst of Pd/MgAl-LDHs exhibited a lower intensity pattern except for the diffraction peaks at 38° and 44°, which was due to the random dispersion of the Pd component on the Pd/MgAl-LDHs surface. The Pd loading of catalyst was 8.5 wt %, and the molar ratio of Mg and Al in LDH layers were 2.97, which is in accordance with the ratio of 3.00 employed in the synthesis step (see Table S1 in the Supporting Information File 1). Furthermore, the binding energy of Pd 3d_5/2_ and Pd 3d_3/2_ in LDH layers approximately centered at 334.7 eV and 340.2 eV, respectively, assigning to the existence zero oxidation state of Pd bulk (around 335.0 eV and 341.0 eV [49–50]), verified that PdCl_4_^2−^ had been reduced to zero-valence Pd from interlayers and loaded on MgAl-LDH surface successfully (see Figure S1 in Supporting Information File 1).

### The Heck coupling reaction under HSBM conditions

**Scheme 2:**
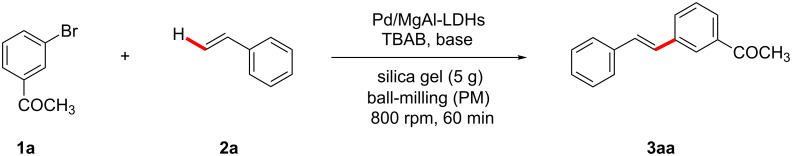
Selected model reaction.

*m*-Bromoacetophenone (**1a**) and styrene (**2a**) were chosen as the model reactants (Scheme 2), catalyzed by Pd/MgAl-LDHs under ball-milling conditions with silica gel (5 g) and stainless-steel balls (Φ_MB_ = 0.2, *d*_MB_ = 14 mm) at 800 rpm.

Based on our previous researches [4,51], it is found that the bases used have a significant influence on the yields of the reaction. Thus, several bases such as NaOH, KOH, Cs_2_CO_3_, K_2_CO_3_, *t*-BuOK, Et_3_N and DBU were investigated and the results are shown in Table 1. It is notable that both inorganic and organic bases could facilitate the reaction successfully. K_2_CO_3_ exhibited the best yield of 72% (Table 1, entries 1–7) as compared with other bases for the reaction. In further tests, different loadings of Pd/MgAl-LDHs were employed in the model reaction (Table 1, entries 8–10) in order to optimize the usage of catalyst. The results show that the reaction yield kept unchanged when the Pd/MgAl-LDHs loading was reduced to 2.5 mol % (Table 1, entry 9).

**Table 1:** Optimization of Heck reaction conditions.^a^

Entry	Base	Pd (mol %)	Yield (%)^b^

1	NaOH	10	56
2	KOH	10	64
3	*t*-BuOK	10	59
4	Et_3_N	10	54
5	Cs_2_CO_3_	10	60
6	K_2_CO_3_	10	72
7	DBU	10	43
8	K_2_CO_3_	5	71
9	K_2_CO_3_	2.5	71
10	K_2_CO_3_	1.25	54

^a^Reaction conditions unless otherwise noted: **1a** (1.5 mmol), **2a** (2.1 mmol), Pd/MgAl-LDHs, TBAB (1.5 mmol), base (3.6 mmol), and 5 g silica gel were placed in a 80 mL stainless-steel vessel (Φ_MB_ = 0.2, *d*_MB_ = 14 mm). HSBM conditions: 60 min at 800 rpm. ^b^Isolated yield.

After getting access to the optimal reactant system, we shifted our focus on the mechanochemistry parameters of mill-ball size and its filling degree. The milling-ball filling degree (Φ_MB_) represents the volume of the milling balls relative to the beaker volume, which is calculated as the ratio of the overall milling ball volume (*V*_MB_) to the total beaker volume (*V*_BV_):


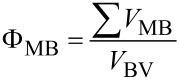


**Figure 2:**
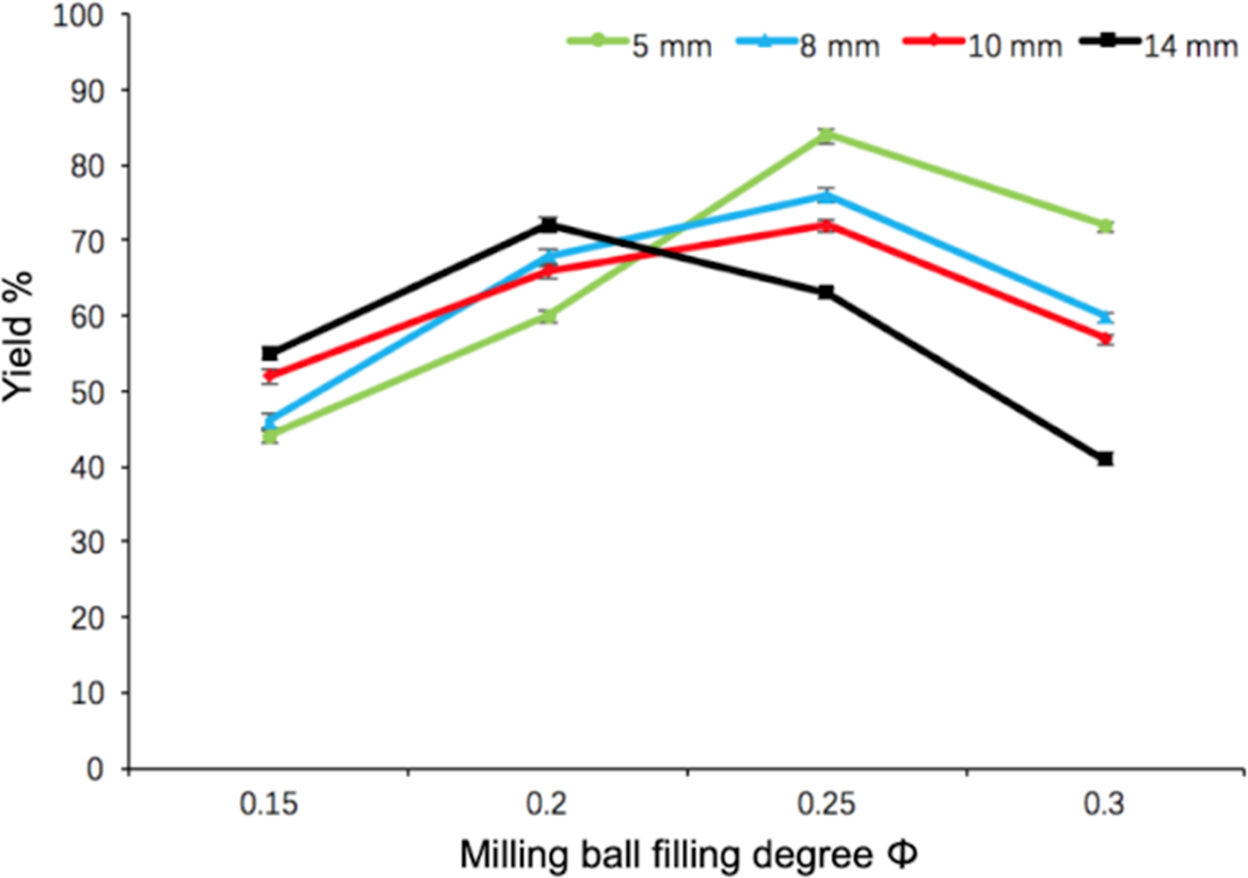
Examination of the milling-ball filling degree (Φ_MB_) and milling-ball sizes on the yield of **3aa**. Reaction conditions: **1a** (1.5 mmol), **2a** (2.1 mmol), Pd/MgAl-LDHs (2.5 mol %), TBAB (1.5 mmol), K_2_CO_3_ (3.6 mmol), 5 g silica gel were placed in a 80 mL stainless-steel vessel. HSBM conditions: 60 min at 800 rpm.

This parameter is proved to be the essential factor not only on the occurrence of collision and friction, but also on the energy distribution and product yield [52]. In Figure 2, we chose four types of milling-balls with diameters of 5 mm, 8 mm, 10 mm and 14 mm in the model reaction under four kinds of filling degrees (Φ_MB_ = 0.15, 0.2, 0.25, 0.3). It could be found that no matter which kind of the milling-ball diameter is, the tendency of the product yield is similar under different filling degrees. A maximum yield (84%) was obtained by using 5 mm milling balls at 0.25 filling degree. In addition, the 14 mm milling balls exhibited the higher yields than 5 mm, 8 mm and 10 mm milling balls under the low filling degrees (Φ_MB_ = 0.15, 0.2). And then, with the filling degree increased to the value of 0.25, the movement space for 14 mm milling balls was hindered in the ball-milling jar, resulting in the apparent decrease in the yield of **3aa**. On the contrary, 5 mm, 8 mm and 10 mm milling balls had sufficient collision to produce enough energy under the filling degree of 0.25, leading to the high yields. Furthermore, the sharp decrease in the yield could be also observed in 5 mm, 8 mm and 10 mm milling-ball systems with a filling degree over 0.25, which might be due to the overfull ball-milling jar and the overabundant energy input. These results mentioned above are consistent with the previous studies reported by us [53] and the others [52,54].

**Figure 3:**
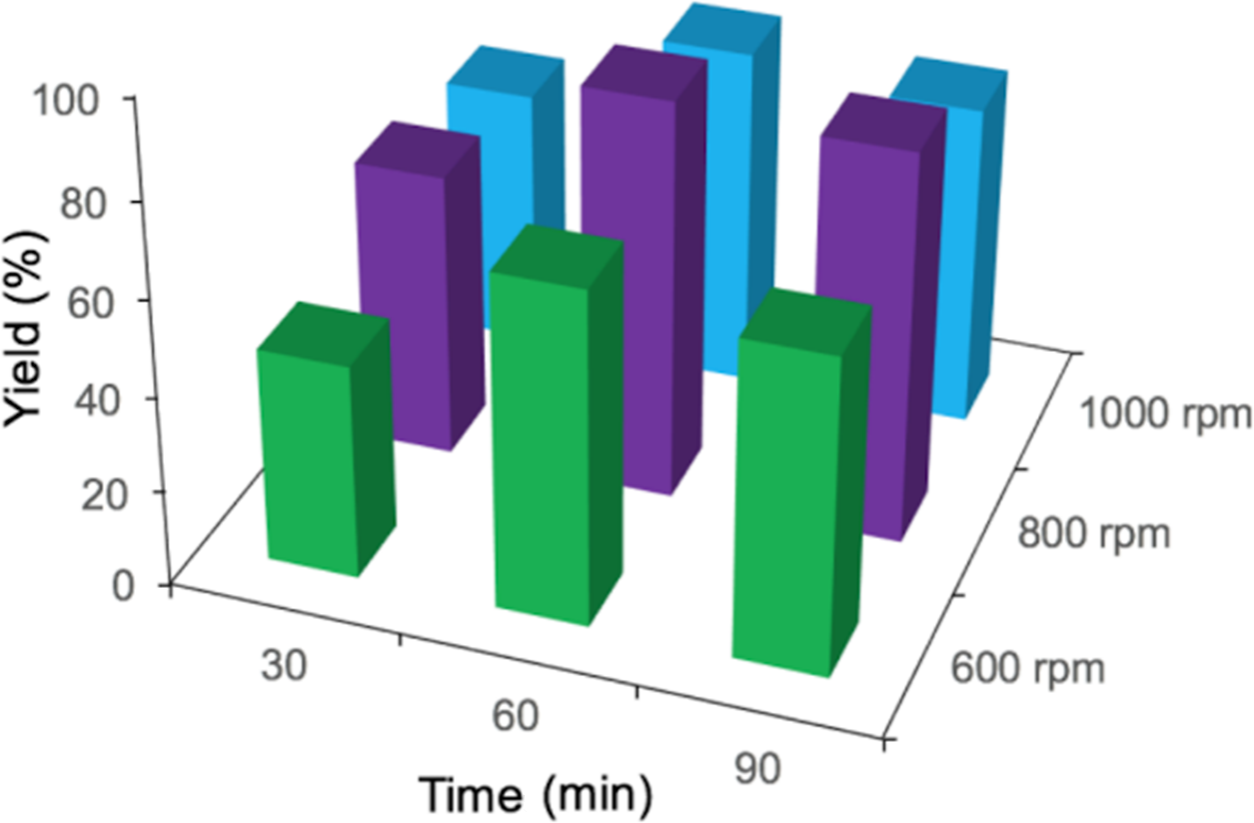
Examination of ball-milling time and rotation speed on the yield of **3aa**. Reaction conditions: **1a** (1.5 mmol), **2a** (2.1 mmol), Pd/MgAl-LDHs (2.5 mol %), TBAB (1.5 mmol), K_2_CO_3_ (3.6 mmol), and 5 g silica gel were placed in a 80 mL stainless-steel vessel (Φ_MB_ = 0.25, *d*_MB_ = 5 mm). HSBM conditions: 60 min at 800 rpm.

Because the ball-milling time and the rotation speed have a strong inﬂuence on the energy input, which directly regulates the product structure and yield during the mechanochemical process, the combined effect of ball-milling time and rotation speed was investigated systematically. The results are summarized in Figure 3. It can be seen that with increasing rotation speed, the yield of **3aa** increased first, but decreased at the highest speed of 1000 rpm. This is mainly due to the overabundant energy input resulting in side products. Furthermore, prolonging the reaction time over 60 min did not help improving the product yield, the reactants had all been consumed after 60 min. Therefore, 800 rpm together with 60 min is regarded as the optimum condition for the maximum yield.

In the ball-milling process, the grinding auxiliary is found to be an efficient transfer medium between energy and reactant [1–2,55–56]. Additional investigations on the effects of the grinding auxiliaries were carried out. The results shown in Table 2 indicate that 5 g silica gel is considered as the most effective choice for the reaction (Table 2, entry 1), but MgAl-LDHs gave also a good result (Table 2, entry 5). With NaCl, α-Al_2_O_3_ and γ-Al_2_O_3_, the yields were unsatisfactory (Table 2, entries 2–4). Increasing or decreasing the amount of silica gel would led to a reduction of the yield of **3aa** (Table 2, entries 6 and 7), which might be due to the uneven distribution of the reactants.

**Table 2:** Examination of grinding auxiliaries on yield of **3aa**.^a^

Entry	Grinding auxiliary	Weight (g)	Yield (%)

1	silica-gel	5	84 (n.r.)^b^
2	NaCl	5	54
3	α-Al_2_O_3_ (base)	5	68
4	γ-Al_2_O_3_(neutral)	5	61
5	MgAl-LDHs	5	72 (n.r.)^c^
6	silica-gel	3	74
7	silica-gel	7	70

^a^Reaction conditions unless otherwise noted: **1a** (1.5 mmol), **2a** (2.1 mmol), Pd/MgAl-LDHs (2.5 mol %), TBAB (1.5 mmol), K_2_CO_3_ (3.6 mmol), grinding auxiliary were placed in a 80 mL stainless-steel vessel (Φ_MB_ = 0.25, *d*_MB_ = 5 mm). HSBM conditions: 60 min at 800 rpm. ^b^Silica gel used as grinding auxiliary without Pd/MgAl-LDHs catalyst. ^c^MgAl-LDHs used as grinding auxiliary without Pd/MgAl-LDHs catalyst.

**Figure 4:**
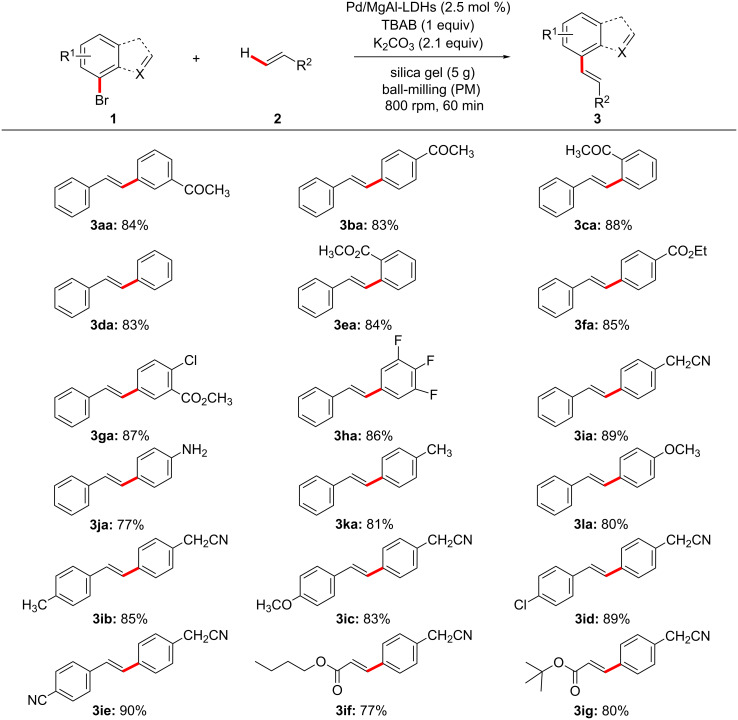
Substrate scope of Pd/MgAl-LDHs catalyzed Heck reactions. Reaction conditions unless otherwise noted: **1a** (1.5 mmol), **2a** (2.1 mmol), Pd/MgAl-LDHs (2.5 mol %), TBAB (1.5 mmol), K_2_CO_3_ (3.6 mmol), and 5 g silica gel were placed in a 80 mL stainless-steel vessel (Φ_MB_ = 0.25, *d*_MB_ = 5 mm). HSBM conditions: 60 min at 800 rpm.

**Scheme 3:**
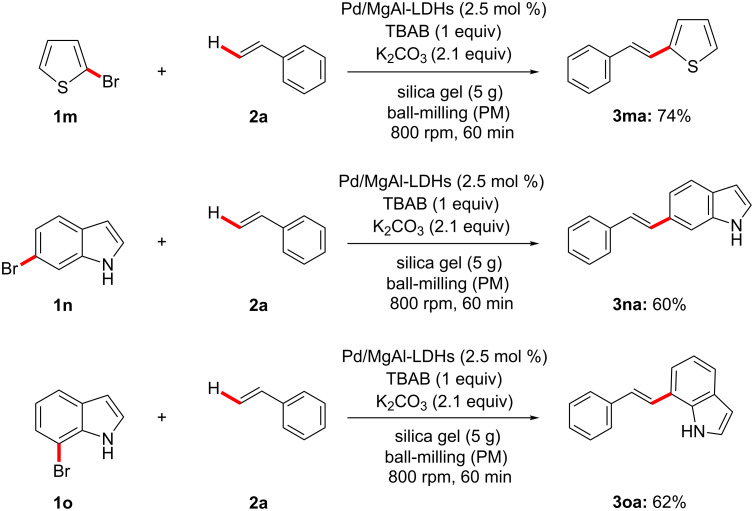
Pd/MgAl-LDHs catalyzed Heck reactions of heteroaryl bromides. Reaction conditions unless otherwise noted: **1a** (1.5 mmol), **2a** (2.1 mmol), Pd/MgAl-LDHs (2.5 mol %), TBAB (1.5 mmol), K_2_CO_3_ (3.6 mmol), and 5 g silica gel were placed in a 80 mL stainless-steel vessel (Φ_MB_ = 0.25, *d*_MB_ = 5 mm). HSBM conditions: 60 min at 800 rpm.

After having the optimum reaction conditions in hand, the Pd/MgAl-LDH catalyst was evaluated to expand the generality and substrate scope in Heck reactions, the results are presented in Figure 4 and Scheme 3. As we expected, both with electron-withdrawing and electron-donating groups substituted bromobenzenes (**1a**–**l**) and styrenes (**2a**–**e**) react with each other successfully to afford the coupling products in satisfactory yields. The electron-deﬁcient bromobenzenes (**1a**–**c**, **1e**–**i**) or styrenes (**2d**, **2e**) show slightly higher yields than the electron-rich substrates. The ketone group at *ortho-*, *meta-* and *para-*positions (**1a**–**c**) were chosen to examine the steric hindrance for this reaction. To our surprise, the position of the ketone group had a little effect on the yields and the larger sterically hindered substrate **1c** led to a higher yield as compared with **1a** and **1b**, which is contrary to Li’s study [57] in solution-based Heck reactions. This might be because of the lone pairs of the oxygen atom in the keto group at the *ortho*-position could coordinate with Pd/MgAl-LDHs under HSBM conditions and promote the reaction efficiently. Furthermore, the couplings of heteroaryl bromides (**1m**–**o**) and styrene (**2a**) as well as substituted bromobenzene (**1i**) and butyl acrylate (**2f**, **2g**) were investigated to extend the scope and generality of the reaction. The results clearly demonstrate that all the substrates are well tolerated to give the corresponding coupling products smoothly with yields of 60–80%.

**Figure 5:**
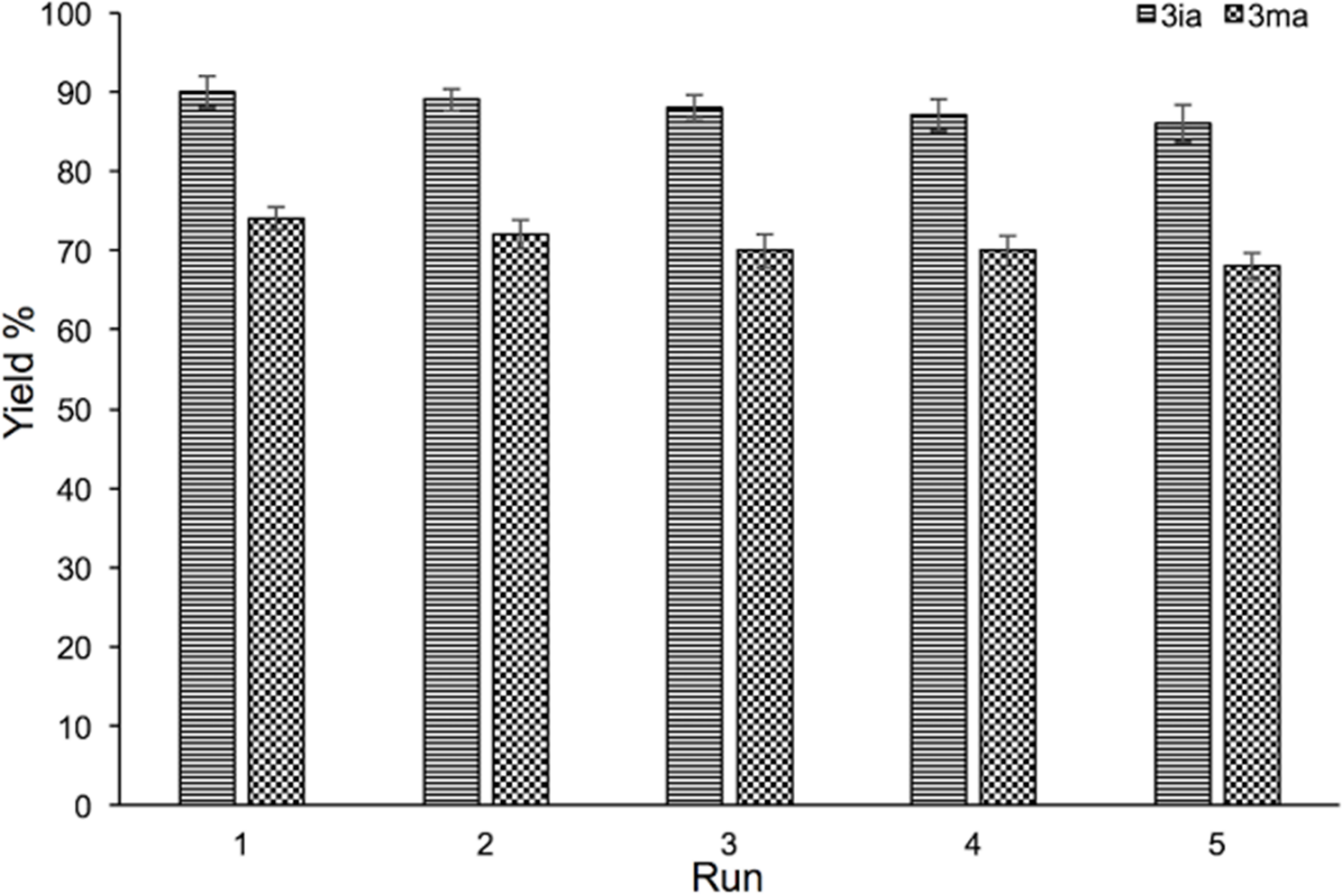
Recycling studies of the Pd/MgAl-LDH catalyst for Heck reactions. Reaction conditions: **1i** or **1m** (1.5 mmol), **2a** (2.1 mmol), Pd/MgAl-LDHs (2.5 mol %), TBAB (1.5 mmol), K_2_CO_3_ (3.6 mmol), and silica gel 5 g were placed in a 80 mL stainless-steel vessel (Φ_MB_ = 0.25, *d*_MB_ = 5 mm). HSBM conditions: 60 min at 800 rpm.

Finally, the coupling reactions of aryl bromide **1i** and styrene (**2a**) as well as heterocyclic bromide **1m** and styrene (**2a**) were chosen as the model reactions under the optimized conditions to investigate the reusability of the Pd/MgAl-LDH catalyst. The catalyst together with the grinding auxiliary are recovered by a simple rinse after each run, which is more convenient compared to other methods [36–37]. As can be seen in Figure 5, regardless of the substrate type, the catalyst system could be reused at least five times efficiently without signiﬁcant loss in catalytic activity, resulting in almost no change in the yields. Hence, the reusability of Pd/MgAl-LDHs is one of the major advantages for Heck reactions under HSBM conditions.

## Conclusion

In summary, a supported and recyclable Pd catalyst (Pd/MgAl-LDHs) was designed and synthesized by co-precipitation and reduction methods. The catlyst was further applied to Heck reactions under HSBM conditions. The results indicate that the Pd is successfully dispersed on the surface of Pd/MgAl-LDHs, and a small quantity of Pd/MgAl-LDHs (2.5 mol % of Pd) shows the remarkable activity in Heck reactions with a wide range of aryl bromides and olefins under mild conditions. In these cases, toxic solvents, expensive ligands and inert atmosphere were efficiently avoided. Furthermore, the Pd/MgAl-LDH catalyst can be recycled for at least five times without significant loss in coupling product yields.

## Supporting Information

File 1 Details of experimental procedures and characterization data of prepared compounds, ^1^H, ^13^C NMR, and MS spectra of all coupling compounds.
