# Supplementary material for: Encaging palladium(0) in layered double hydroxide: A sustainable catalyst for solvent-free and ligand-free Heck reaction in a ball mill
Source: Beilstein J Org Chem. 2017 Aug 14;13:1661–8. doi: 10.3762/bjoc.13.160 (PMC5564263; doi:10.3762/bjoc.13.160)
Supplement: File 1 — Details of experimental procedures and characterization data of prepared compounds, 1H, 13C NMR, and MS spectra of all coupling compounds. [file Beilstein_J_Org_Chem-13-1661-s001.pdf]

Supporting Information

for

Encaging palladium(0) in layered double hydroxide: A sustainable catalyst for solvent-free and ligand-free Heck reaction in a ball mill

Wei Shi, Jingbo Yu, Zhijiang Jiang, Qiaoling Shao and Weike Su*

Address: National Engineering Research Center for Process Development of Active Pharmaceutical Ingredients, Collaborative Innovation Center of Yangtze River Delta Region Green Pharmaceuticals, Zhejiang University of Technology, Hangzhou, 310014, Zhejiang, China

Email: Weike Su - pharmlab@zjut.edu.cn

* Corresponding author

Details of experimental procedures and characterization data of prepared compounds, ^{1}H, ^{13}C NMR, and MS spectra of all coupling compounds

Table of Content

1. Experimental section **S2**

1.1 Materials **S2**

1.2 Method **S3**

1.3 Preparation of Pd/MgAl-LDHs **S3**

1.3.1 Synthesis of MgAl-LDHs S3

1.3.2 Synthesis of MgAl-LDHs-$PdCl_4^{2-}$ S4

1.3.3 Synthesis of Pd/MgAl-LDHs S4

1.4 General procedure of Heck reaction and catalyst recycling S4

2. Characterization of Pd/MgAl-LDHs S5

3. Characterization data of 3 .... S6

4. ^{1}H, ^{13}C NMR spectra of 3 .... S13

5. References S39

1. Experimental section

1.1 Materials

Magnesium nitrate hexahydrate ($Mg(NO_3)_2{\cdot}6H_2O$), aluminium nitrate nonahydrate ($Al(NO_3)_3{\cdot}9H_2O$), sodium hydroxide (NaOH), Na_2PdCl_4, $N_2H_4{\cdot}H_2O$ and ethanol were analytical reagent (AR) and purchased from Aladdin Chemistry Co., Ltd. All aryl bromides and olefinic derivatives used for the Heck coupling reactions were purchased from Energy Chemical Co., Ltd. All the above chemicals were used as purchased without further purification. Besides that, decarbonated water was prepared by boiling deionized water to avoid contamination with carbonate anions before employing in synthetic process of Pd/MgAl-LDHs.

1.2 Method

Power X-ray diffraction (XRD) measurements were recorded on a X'pert pro diffractometer (PANalytical Corporation, Netherlands), operating on Cu Kα radiation (λ = 0.1542 nm) with the voltage and anode current of 40 kV and 40 mA at room temperature. The scanning speed was in steps of 5°/min over the angular variation range of 5–80 (2θ). The Pd loading as well as the content of Mg, Al element in Pd/MgAl-LDHs were determined by using inductively coupled plasma mass spectrometry (ICP–MS) on an Elan DRC-e instrument (PerkinElmer Corporation, USA). X-ray photoelectron spectroscopy (XPS) studies were carried out on a Kratos Axis Ultra DLD spectrometer (Kratos Corporation, UK) with the help of monochromatic Al Kα excitation (1486.6 eV) operated at 45 W. The peak of C 1*s* at 284.6 eV was employed to calibrate the binding energies. All coupling products were purified by flash column chromatography on silica gel. Melting points (mp) were measured on a digital melting point instrument. ^{1}H and ^{13}C NMR spectra were recorded using $CDCl_3$ as the solvent at 400 (or 500) MHz and 100 MHz respectively, and TMS was used as the internal standard. Mass spectra were measured with a HRMS-ESI-Q-TOF and a low-resolution MS instrument using an ESI ion source.

1.3 Preparation of Pd/MgAl-LDHs

1.3.1 Synthesis of MgAl-LDHs

The MgAl-LDHs by fixing the molar ratio of Mg:Al at 3:1 was prepared by using co-precipitation route under nitrogen atmosphere as described below: A 80mL aqueous solution containing 0.2 mol (8.0 g) NaOH and a 80mL aqueous solution containing 0.075 mol (19.2 g) $Mg(NO_3)_2\cdot 6H_2O$ and 0.025 mol (9.4 g) $Al(NO_3)_3\cdot 9H_2O$ were added dropwise to a flask (500 mL) with 80 mL decarbonated water under vigorous stirring at 25 °C. At the same time, the pH of mixed solution was kept constant at 9–10 and stirred strongly for 45 min.

After that, the resulting mixture was aged at 65 °C for 18 h, filtered and rinsed several times with deionized water until pH reached 7. Finally, it was dried in vacuum oven for 18 h at 65 °C to obtained the product of MgAl-LDHs.

1.3.2 Synthesis of MgAl-LDHs-$PdCl_4^{2-}$

MgAl-LDHs-$PdCl_4^{2-}$ was prepared by use of anionic exchange reaction. For this reason, the previously synthesized MgAl-LDHs (3.0 g) was added to 0.833 g of Na_2PdCl_4 in 300 mL of decarbonated water under nitrogen atmosphere and maintained with constant stirring for 12 h at 25 °C. The brown product of MgAl-LDHs-$PdCl_4^{2-}$ was isolated by centrifugation, and washed with distilled water for five times, subsequently dried overnight in vacuum oven at 60 °C.

1.3.3 Synthesis of Pd/MgAl-LDHs

A reduction process was chosen to prepare Pd/MgAl-LDHs as shown below: 50 mL of ethanol was added to 6.0 g dried power of MgAl-LDHs-$PdCl_4^{2-}$. Then the ethanol solution of MgAl-LDHs-$PdCl_4^{2-}$ was reduced by $N_2H_4 \cdot H_2O$ (5 mL) for 3 h at 25 °C. Finally, the catalyst was centrifuged and washed with ethanol, and vacuum-dried under 60 °C for 12 h.

1.4 General procedure of Heck reaction and catalyst recycling

In a mechanically assisted Heck reaction, Pd/MgAl-LDHs (2.5 mol%) was added to a ball-milling jar (80 mL) together with aryl bromides (1.5 mmol), olefinic derivatives (2.1 mmol), potassium carbonate (K_2CO_3) (3.6 mmol), tetra-*n*-butylammonium bromide (TBAB) (1.5 mmol) and silica gel (5.0 g). After that, designated amount (Φ_{MB} = 0.25) of stainless steel balls with d_{MB} of 5 mm were added and the jar was locked with a lid and gasket. The reaction mixture

was then ball-milled with rotational speed of 800 rpm for 60 min. After end up of the reaction, all the mixture was dissolved by using ethyl acetate (EtOAc) (50 mL) and centrifugated to get the filtrate and residue. The filtrate was dried with anhydrous sodium sulfate (Na_2SO_4) and concentrated by rotary evaporation. The products were purified by column chromatography (petroleum ether/ethyl acetate). After each reaction, the residue was washed with decarbonated water, ethanol and EtOAc three times to obtain the mixture of catalyst and silica gel, which was vacuum-dried to applied in Heck reaction for the next run.

2. Characterization of Pd/MgAl-LDHs

Table S1 The metallic composition of Pd/MgAl-LDHs

Catalyst	Mg (wt %)	Al (wt %)	Pd (wt %)
Pd/MgAl-LDHs	33.9	12.8	8.5

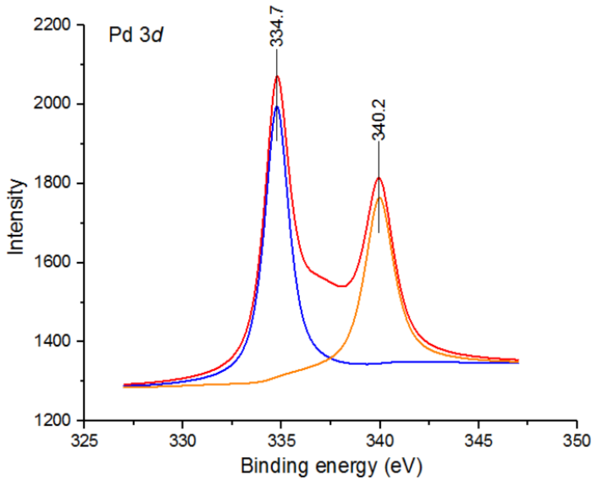

Figure S1. XPS spectra of Pd 3d for Pd/MgAl-LDHs (red line), Pd $3d_{5/2}$ (blue line), Pd $3d_{3/2}$ (yellow line)

3. Characterization data of 3

3aa: (*E*)-1-(3-Styrylphenyl)ethanone

White solid; mp: 71-72 °C (lit 70-74 °C [1]); ^{1}H NMR (400 MHz, $CDCl_3$): δ 8.07(s, 1H), 7.81 (d, *J* = 8.0 Hz, 1H), 7.67 (d, *J* = 8.0 Hz, 1H), 7.50 (d, *J* = 8.0 Hz, 2H), 7.45-7.33 (m, 3H), 7.27 (d, *J* = 8.0 Hz, 1H), 7.19-7.09 (m, 2H), 2.63 (s, 3H); ^{13}C NMR (100 MHz, $CDCl_3$): δ 197.5, 138.0, 137.8, 137.1, 130.7, 130.2, 128.8 (2C), 128.7, 128.0, 127.7, 126.3, 126.7 (2C), 126.2, 26.7; MS (ESI) 223.2 ($[M + H]^+$).

3ba: (*E*)-4-Acetylstilbene

White solid; mp: 140-142 °C (lit 141-142 °C [2]); ^{1}H NMR (500 MHz, $CDCl_3$): δ 7.95 (d, *J* = 10.0 Hz, 2H), 7.59 (d, *J* = 5.0 Hz, 2H), 7.54(d, *J* = 10.0 Hz, 2H), 7.38 (t, *J* = 7.5 Hz, 2H), 7.31 - 7.29 (m, 1H), 7.25-7.11 (m, 2H), 2.61 (s, 3H); ^{13}C NMR (100 MHz, $CDCl_3$): δ 197.2, 142.0, 136.7, 136.0, 131.5, 128.9 (2C), 128.8 (2C), 128.3 (2C), 127.5, 126.8 (2C), 126.5, 26.8; MS (ESI) 223.1 ($[M + H]^+$).

3ca: (*E*)-1-(2-Styrylphenyl)ethanone

Colourless oil [3]; ^{1}H NMR (400 MHz, $CDCl_3$): δ 7.68-7.62 (m, 3H), 7.51-7.47 (m, 2H), 7.45 (td, J = 8.0, 0.8 Hz, 1H), 7.35-7.28 (m, 3H), 7.26-7.22 (m, 1H), 6.95(d, J = 16.0 Hz, 1H), 2.59 (s, 3H); 13C NMR (100 MHz, CDCl3): δ 202.0, 137.3 (2C), 137.2, 131.6, 131.5, 129.1, 128.6 (2C), 127.8, 127.4, 127.3 (2C), 127.2, 126.8, 30.1; MS (ESI) 223.1 ($[M + H]^+$).

3da: (*E*)-1,2-Diphenylethene

White solid; mp: 120-121 °C (lit 120-121 °C [4]) ;^{1}H NMR (400 MHz, $CDCl_3$) δ 7.49 (d, *J* = 8.0 Hz, 4H), 7.34 (t, *J* = 8.0 Hz, 4H), 7.26-7.22 (m, 2H), 7.10 (s, 2H); ^{13}C NMR (100 MHz, $CDCl_3$): δ 137.3 (2C), 128.7 (6C), 127.6 (2C), 126.5 (4C); MS (ESI) 181.1 ($[M + H]^+$).

3ea: (*E*)-Methyl 2-[2-phenylethenyl]benzoate

Yellow oil [5]; ^{1}H NMR (400 MHz, $CDCl_3$) δ 7.96 (d, *J* = 16.0 Hz, 1H), 7.90 (d, *J* = 8.0 Hz, 1H), 7.69 (d, *J* = 8.0 Hz, 1H), 7.52 (d, *J* = 8.0 Hz, 2H), 7.47 (d, *J* = 8.0 Hz, 1H), 7.36-7.22 (m, 4H), 6.98 (d, *J* = 16.0 Hz, 1H), 3.91 (s, 3H); ^{13}C NMR (100 MHz, $CDCl_3$): 167.8, 139.2, 137.4, 134.3, 132.1, 131.5, 130.7, 128.7 (2C), 127.9, 127.5, 127.1, 127.0 (2C), 126.9, 52.3; MS (ESI) 239.1 ($[M + H]^+$).

3fa: (*E*)-4-Ethoxycarbonylstilbene

Colorless solid; mp: 106-107 °C (lit 106-106.5 °C [6]); ^{1}H NMR (500 MHz, $CDCl_3$): δ 8.02 (d, *J* = 8.0 Hz, 2H), 7.57-7.52 (m, 4H), 7.42-7.39 (m, 2H), 7.33-7.30 (m, 1H), 7.21 (d, *J* = 16.5 Hz, 1H), 7.12 (d, *J* = 16.5 Hz, 1H), 4.41-4.35 (m, 2H), 1.40 (t, *J* = 7.0 Hz, 3H); ^{13}C NMR (100 MHz, $CDCl_3$): δ 166.3, 141.7, 136.8, 131.2, 130.0 (2C), 129.4, 128.8 (2C), 128.2, 127.7 (2C), 126.8 (2C), 126.3, 61.1, 14.7; MS (ESI) 253.2 ($[M + H]^+$).

3ga: (*E*)-Methyl 2-chloro-5-styrylbenzoate

Colourless oil; ^{1}H NMR (500 MHz, $CDCl_3$): δ 7.94 (d, *J* = 2.5 Hz, 1H), 7.53-7.49 (m, 3H), 7.41 (d, *J* = 8.0 Hz, 1H), 7.36 (t, *J* = 7.5 Hz, 2H), 7.30-7.27 (m, 1H), 7.12 (d, *J* = 16.0 Hz, 1H), 7.03 (d, *J* = 16.0 Hz, 1H), 3.95 (s, 3H); ^{13}C

NMR (100 MHz, $CDCl_3$): δ (100 MHz, $CDCl_3$): 166.2, 136.7, 136.2, 132.4, 131.4, 130.6, 130.3, 130.1, 129.4 (2C), 128.9, 128.3, 126.8, 126.4 (2C), 52.6; HRMS (ESI) $C_{16}H_{13}NaClO_2$ ($[M + Na]^+$) calcd 295.0496, found 295.0488.

3ha: (*E*)-3,4,5-Trifluorostilbene

Colourless oil [1]; ^{1}H NMR (400 MHz, $CDCl_3$): δ 7.45 (d, *J* = 7.6 Hz, 2H), 7.36-7.25 (m, 3H), 7.07-7.02 (m, 2H), 6.97 (d, *J* = 16.4 Hz, 1H), 6.87 (d, J = 16.0 Hz, 1H); ^{13}C NMR (100 MHz, $CDCl_3$): δ 152.6 (2C), 150.1, 136.2, 133.7, 131.1, 128.8 (2C), 128.4, 126.7(2C), 125.7, 110.1 (2C); MS (ESI) 235.1 ($[M + H]^+$).

3ia: (*E*)-2-(4-Styrylphenyl)acetonitrile

White solid; mp: 121-122 °C (lit 121.6-122.5 °C [1]); ^{1}H NMR (400 MHz, $CDCl_3$): δ 7.49 (d, *J* = 8.0 Hz, 4H), 7.35 (t, *J* = 7.6 Hz, 2H), 7.31-7.23 (m, 3H), 7.13-7.03 (m, 2H), 3.74 (s, 2H); ^{13}C NMR (100 MHz, $CDCl_3$): δ 137.2, 136.9, 129.5, 128.9, 128.7 (2C), 128.3, 127.9 (2C), 127.6 (2C), 127.1 (2C), 126.6, 117.8, 23.7; MS (ESI) 220.2 ($[M + H]^+$).

3ja: (*E*)-4-Styrylaniline

Yellow solid; mp: 146-147 °C (lit 147-148 °C [4]); ^{1}H NMR (500 MHz, $CDCl_3$): δ 7.46 (d, *J* = 7.5 Hz, 2H), 7.35-7.31 (m, 4H), 7.26-7.22 (m, 1H), 7.02 (d, *J* = 16.0 Hz, 1H), 6.92 (d, *J* = 16.0 Hz, 1H), 6.68 (d, *J* = 8.5 Hz, 2H), 3.75 (bs, 2H); ^{13}C NMR (100 MHz, $CDCl_3$): δ 146.3, 138.1, 128.8 (2C), 128.7, 128.2 (2C), 127.9, 127.0 (2C), 126.2, 125.3, 115.4 (2C); MS (ESI) 196.1 ($[M + H]^+$).

3ka: (*E*)-1-Methyl-4-styrylbenzene

White solid; mp: 117-118 °C (lit 118-119 °C [4]); ^{1}H NMR (400 MHz, $CDCl_3$): δ 7.48 (d, *J* = 6.8 Hz, 2H), 7.39 (d, *J* = 8.0 Hz, 2H), 7.33 (t, *J* = 7.2 Hz, 2H), 7.24-7.20 (m, 1H), 7.14 (d, *J* = 8.0 Hz, 2H), 7.10-7.01 (m, 2H), 2.35 (s, 3H); ^{13}C NMR (100 MHz, $CDCl_3$): δ 137.5, 134.5, 129.4, 128.7 (2C), 128.6 (2C), 127.7, 127.4 (2C), 126.5 (2C), 126.4 (2C), 21.5. MS (ESI) 195.2 ($[M + H]^+$).

3la: (*E*)-1-Methoxy-4-styrylbenzene

Yellow solid; mp: 137-138 °C (lit 135-138 °C [4]); ^{1}H NMR (500 MHz, $CDCl_3$): δ 7.50-7.44 (m, 4H), 7.34 (t, *J* = 7.5 Hz, 1H), 7.26-7.23 (m, 2H), 7.10-6.99 (m, 2H), 6.95 (d, *J* = 9.0 Hz, 1H), 6.90 (d, *J* = 8.5 Hz, 1H), 3.84 (s, 3H); ^{13}C NMR (100 MHz, $CDCl_3$): δ 158.7, 137.7, 133.6, 128.7, 128.3 (2C), 127.8 (2C), 127.2, 126.7 (2C), 126.3, 114.3 (2C), 55.5; MS (ESI) 211.2 ($[M + H]^+$).

3ib: (*E*)-2-(4-(4-Methylstyryl)phenyl)acetonitrile

White solid; mp: 173-174 °C; ^{1}H NMR (500 MHz, $CDCl_3$): δ 7.50 (d, *J* = 8.0 Hz, 2H), 7.41 (d, *J* = 8.0 Hz, 2H), 7.30 (d, *J* = 8.0 Hz, 2H), 7.17 (d, *J* = 8.0 Hz, 2H), 7.11 (d, *J* = 16.5 Hz, 1H), 7.06 (d, *J* = 16.0 Hz, 1H), 3.75 (s, 2H), 2.36 (s, 3H); ^{13}C NMR (100 MHz, $CDCl_3$): δ 138.0, 137.6, 134.3, 129.6, 128.8 (2C), 128.4 (2C), 127.1 (2C), 126.8 (2C), 126.7 (2C), 117.9, 23.5, 21.4. HRMS (ESI) $C_{17}H_{16}N$ ($[M + H]^+$) calcd 234.1277, found 234.1265.

3ic: (*E*)-2-(4-(4-Methoxystyryl)phenyl)acetonitrile

White solid; mp: 175-176 °C; ^{1}H NMR (500 MHz, $CDCl_3$): δ 7.50-7.44 (m, 4H), 7.30 (d, *J* = 8.0 Hz, 2H), 7.07 (d, *J* = 16.0 Hz, 1H), 6.95 (d, *J* = 16.5 Hz, 1H),

6.92-6.89 (m, 2H), 3.84 (s, 3H), 3.75 (s, 2H); ^{13}C NMR (100 MHz, $CDCl_3$): δ 159.7, 137.8, 129.9, 129.2, 128.6 (2C), 128.4 (2C), 127.9, 127.0, 125.7 (2C), 117.9, 114.3 (2C), 55.5, 23.5. HRMS (ESI) $C_{17}H_{16}NO$ ([M + H]$^+$) calcd 250.1126, found 250.1129.

3id: (*E*)-2-(4-(4-Chlorostyryl)phenyl)acetonitrile

White solid; mp: 173-174 °C; ^{1}H NMR (500 MHz, $CDCl_3$): 7.51 (d, *J* = 8.5 Hz, 2H), 7.44 (d, *J* = 8.5 Hz, 2H), 7.34-7.31 (m, 4H), 7.06 (s, 2H), 3.76 (s, 2H); ^{13}C NMR (100 MHz, $CDCl_3$): δ 137.1, 135.6, 133.6, 129.3, 129.0 (2C), 128.5 (2C), 128.4 (2C), 128.3, 127.8 (2C), 127.3, 117.8, 23.6; HRMS (ESI) $C_{16}H_{13}NCl$ ([M + H]$^+$) calcd 254.0731, found 254.0738.

3ie: (*E*)-4-(4-(Cyanomethyl)styryl)benzonitrile

Colourless oil; ^{1}H NMR (500 MHz, $CDCl_3$): δ 7.64 (d, *J* = 8.5 Hz, 2H), 7.54 (d, *J* = 8.0 Hz, 2H), 7.41 (d, *J* = 8.5 Hz, 2H), 7.35 (d, *J* = 8.5 Hz, 2H), 7.20 (d, *J* = 16.0 Hz, 1H), 7.11 (d, *J* = 16.5 Hz, 1H), 3.81 (s, 2H); ^{13}C NMR (100 MHz, $CDCl_3$): δ 141.6, 136.4, 132.7 (2C), 131.4, 129.4, 128.6 (2C), 128.5, 127.7 (2C), 127.1 (2C), 119.0, 117.7, 111.0, 23.5; HRMS (ESI) $C_{17}H_{12}N_2Na$ ([M + Na]$^+$) calcd 267.0893, found 267.0887.

3if: (*E*)-Butyl 3-(4-(cyanomethyl)phenyl)acrylate

Colourless oil; ^{1}H NMR (500 MHz, $CDCl_3$): δ 7.65 (d, *J* = 16.0 Hz, 1H), 7.53 (d, *J* = 8.5 Hz, 2H), 7.35 (d, *J* = 8 Hz, 2H), 6.45 (d, *J* = 16.0 Hz, 1H), 4.21 (t, *J* = 6.5 Hz, 2H), 3.78 (s, 2H), 1.72-1.66 (m, 2H), 1.48-1.40 (m, 2H), 0.97 (t, *J* = 7.5 Hz, 3H). ^{13}C NMR (100 MHz, $CDCl_3$): δ 166.8, 143.4, 134.4, 131.9, 128.7 (2C),

128.5 (2C), 119.2, 117.4, 64.6, 30.8, 23.5, 19.2, 13.8. HRMS (ESI) $C_{15}H_{18}NO_2$ ($[M + H]^+$) calcd 244.1332, found 244.1325.

3ig: (*E*)-*tert*-Butyl 3-(4-(cyanomethyl)phenyl)acrylate

Colourless oil [7]; ^{1}H NMR (500 MHz, $CDCl_3$): δ 7.58-7.51 (m, 3H), 7.34 (d, *J* = 8.0 Hz, 2H), 6.38 (d, *J* = 16.0 Hz, 1H), 3.77 (s, 2H), 1.54(s, 9H); ^{13}C NMR (100 MHz, $CDCl_3$): δ 166.2, 142.5, 134.8, 131.6, 128.7 (2C), 128.6 (2C), 121.2, 117.5, 80.9, 28.3 (3C), 23.6; HRMS (ESI) $C_{15}H_{18}NO_2$ ($[M + H]^+$) calcd 244.1332, found 244.1338.

3ma: (*E*)-2-Styrylthiophene

White solid; mp: 106-107 °C (lit 109-110°C [4]); ^{1}H NMR (400 MHz, $CDCl_3$): δ 7.44 (3, *J* = 7.6 Hz, 2H), 7.32 (t, *J* = 7.2 Hz, 2H), 7.24-7.16 (m, 3H), 7.04 (d, *J* = 3.6 Hz, 1H), 6.98 (t, *J* = 3.6 Hz, 1H), 6.91 (d, *J* = 16.0 Hz, 1H); ^{13}C NMR (100 MHz, $CDCl_3$): δ 142.8, 136.9, 128.7, 128.3, 127.6 (2C), 126.3, 126.1 (2C), 124.4 (2C), 121.8; MS (ESI): 187.1 ($[M + H]^+$).

3na: (*E*)-6-Styryl-1*H*-indole

Colourless oil [8]; ^{1}H NMR (400 MHz, $CDCl_3$): δ 8.20-8.06 (brs, 1H), 7.60 (d, *J* = 8.0 Hz, 1H), 7.54-7.46 (m, 3H), 7.38-7.30 (m, 3H), 7.26-7.18 (m, 3H, including 7.22 (d, *J* = 16.0 Hz, 1H)), 7.10 (d, *J* = 16.0 Hz, 1H), 6.53 (s, 1H); ^{13}C NMR (100 MHz, $CDCl_3$, ppm): δ 137.8, 136.2, 131.8, 129.9, 128.7 (2C), 127.8, 127.2, 126.8 (2C), 126.3, 124.9, 120.9, 118.7, 109.6, 102.9; MS (ESI): 220.1 ($[M + H]^+$).

3oa: (*E*)-7-Styryl-1*H*-indole:

Colourless oil [9]; ^{1}H NMR (400 MHz, $CDCl_3$): δ 8.50-8.45 (brs, 1H), 7.62-7.50 (m, 3H), 7.42-7.33 (m, 4H), 7.31-7.22 (m, 2H), 7.21-7.09 (m, 2H), 6.60 (s, 1H); ^{13}C NMR (100 MHz, $CDCl_3$): δ 137.4, 133.7, 129.7, 128.8 (2C), 128.5 (2C), 127.7, 126.4 (2C), 125.1, 124.3, 121.4, 120.5, 120.2, 103.3; MS (ESI): 220.1 ($[M + H]^+$).

4. ^{1}H, ^{13}C NMR spectra of 3

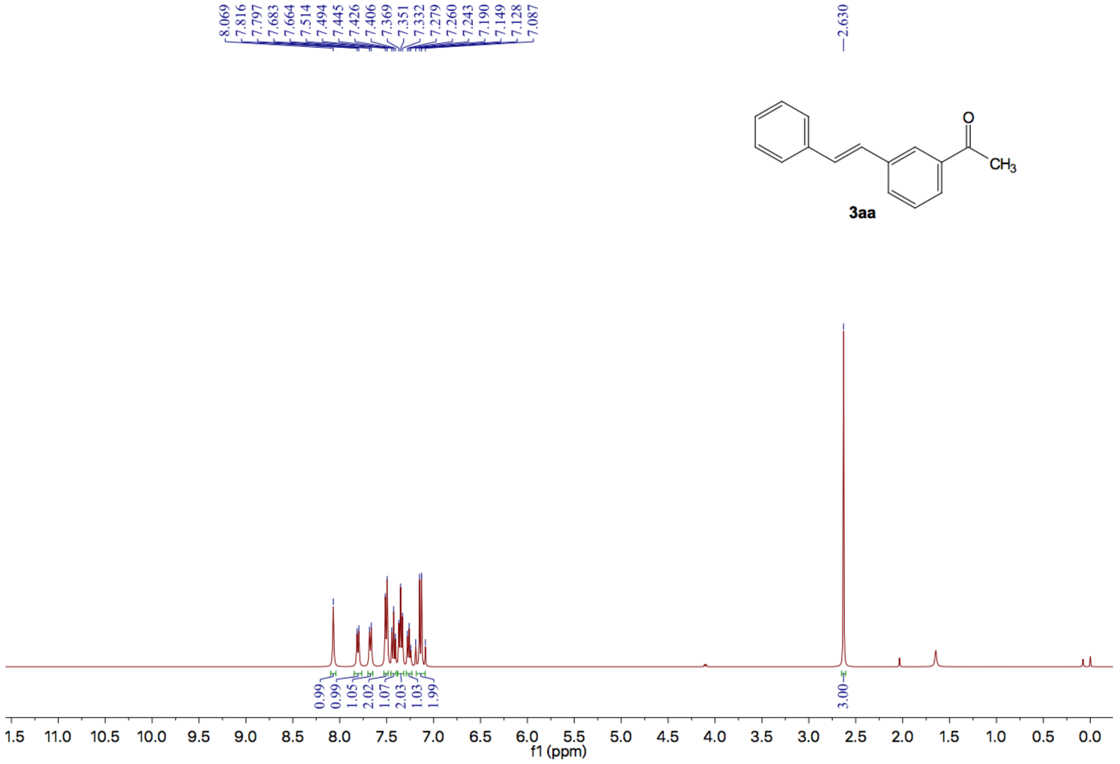

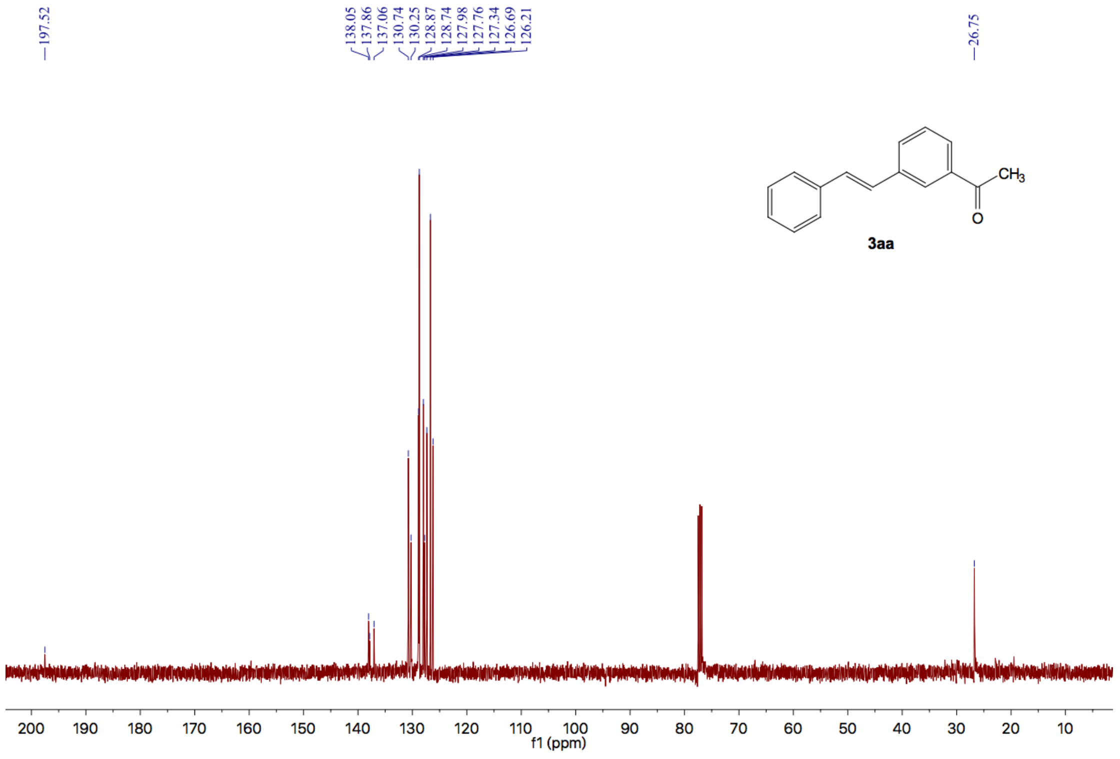

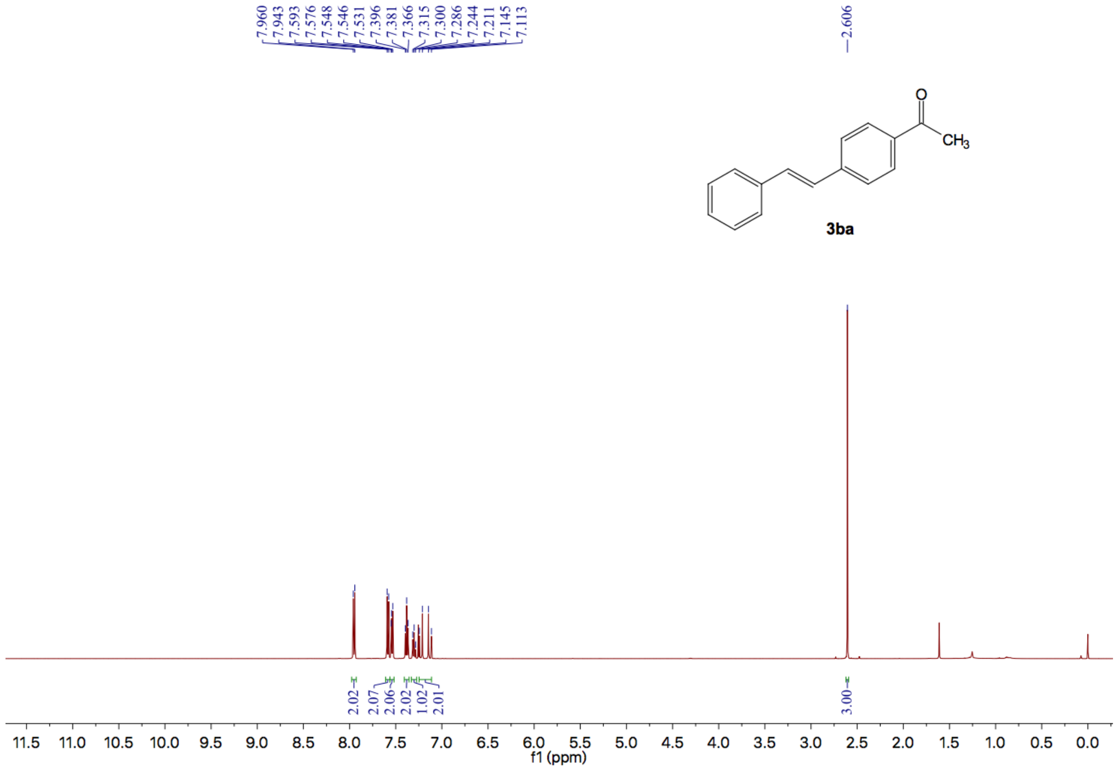

7.960
7.943
7.593
7.576
7.548
7.546
7.531
7.396
7.381
7.366
7.315
7.300
7.286
7.244
7.211
7.145
7.113
2.606
O
CH3
3ba
2.02
2.07
2.06
2.02
1.02
2.01
3.00
11.5 11.0 10.5 10.0 9.5 9.0 8.5 8.0 7.5 7.0 6.5 6.0 5.5 5.0 4.5 4.0 3.5 3.0 2.5 2.0 1.5 1.0 0.5 0.0
f1 (ppm)

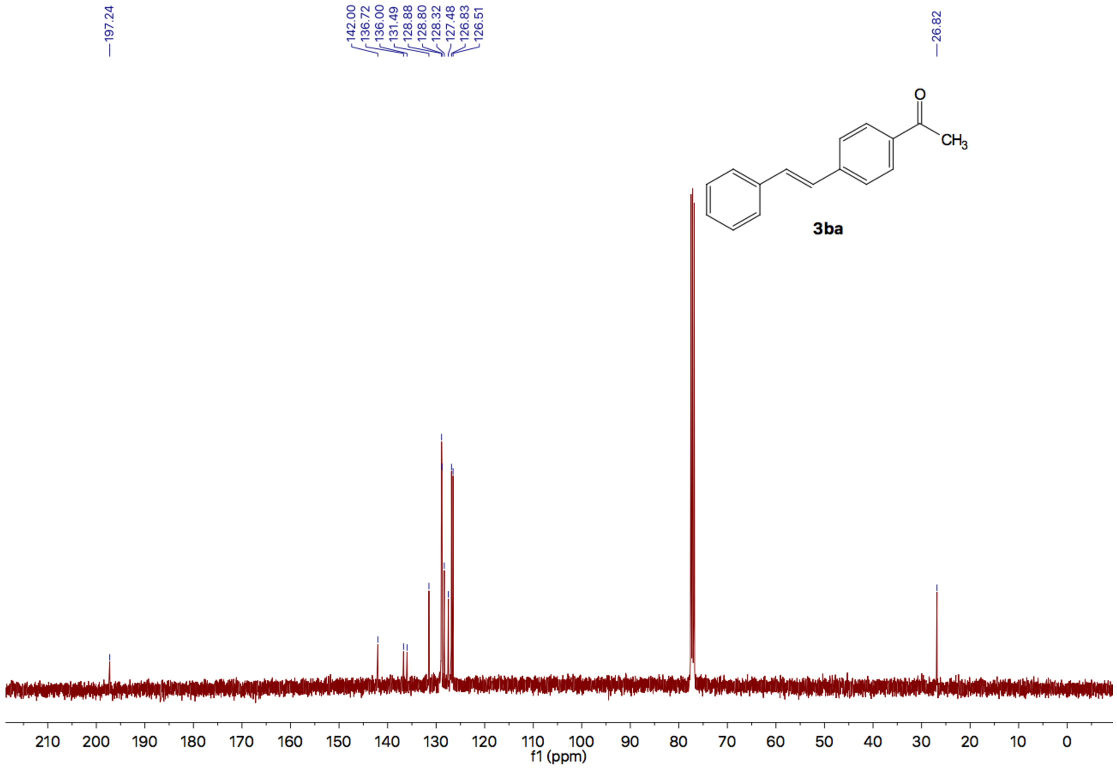

197.24
142.00
136.72
136.00
131.49
128.88
128.80
128.32
127.48
126.83
126.51
26.82
O
CH3
3ba
210 200 190 180 170 160 150 140 130 120 110 100 90 80 70 60 50 40 30 20 10 0
f1 (ppm)

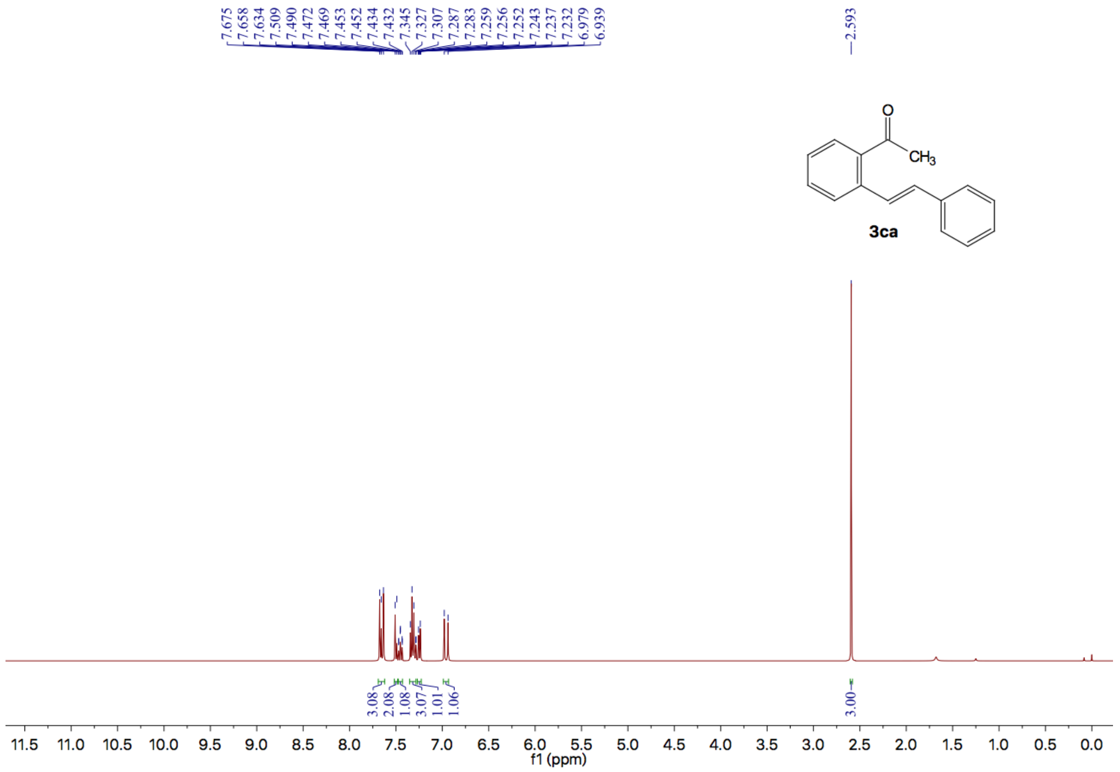
7.675
7.658
7.634
7.509
7.490
7.472
7.469
7.453
7.452
7.434
7.432
7.345
7.327
7.307
7.287
7.283
7.259
7.256
7.252
7.243
7.237
7.232
6.979
6.939
2.593
O
CH3
3ca
3.08
2.08
1.08
3.07
1.01
1.06
3.00
11.5 11.0 10.5 10.0 9.5 9.0 8.5 8.0 7.5 7.0 6.5 6.0 5.5 5.0 4.5 4.0 3.5 3.0 2.5 2.0 1.5 1.0 0.5 0.0
f1 (ppm)

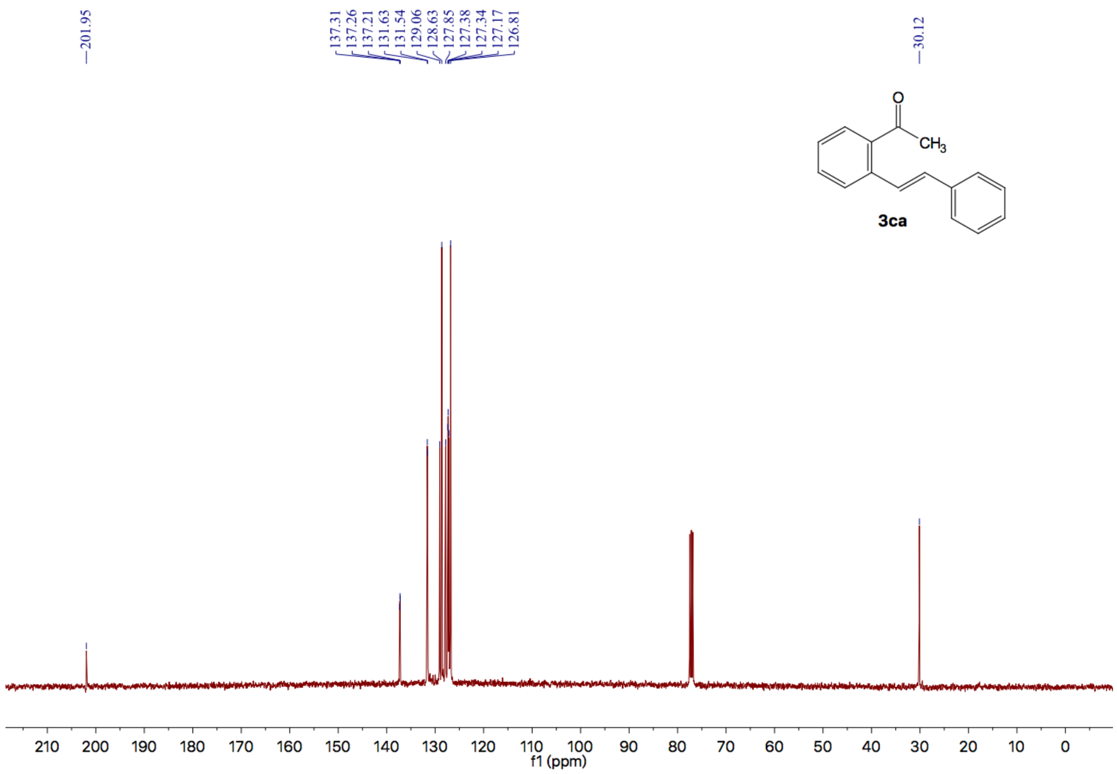
201.95
137.31
137.26
137.21
131.63
131.54
129.06
128.63
127.85
127.38
127.34
127.17
126.81
30.12
O
CH3
3ca
210 200 190 180 170 160 150 140 130 120 110 100 90 80 70 60 50 40 30 20 10 0
f1 (ppm)

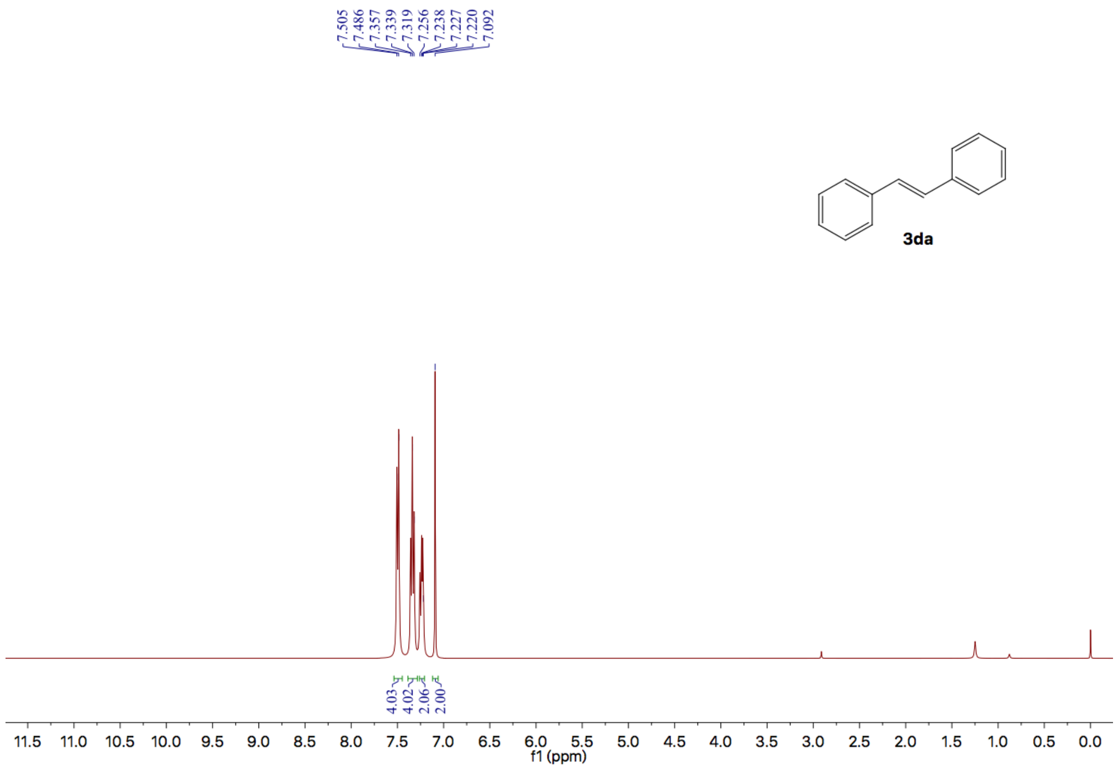
7.505
7.486
7.357
7.339
7.319
7.256
7.238
7.227
7.220
7.092
3da
4.03
4.02
2.06
2.00
11.5 11.0 10.5 10.0 9.5 9.0 8.5 8.0 7.5 7.0 6.5 6.0 5.5 5.0 4.5 4.0 3.5 3.0 2.5 2.0 1.5 1.0 0.5 0.0
f1 (ppm)

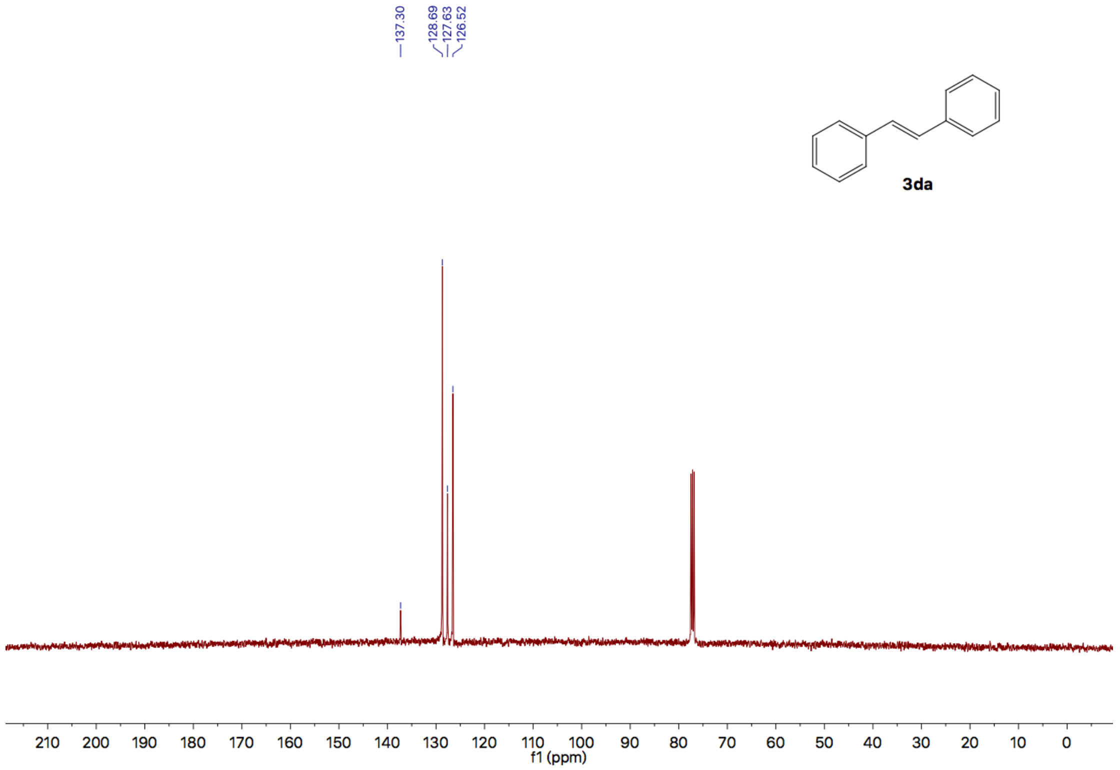
137.30
128.69
127.63
126.52
3da
210 200 190 180 170 160 150 140 130 120 110 100 90 80 70 60 50 40 30 20 10 0
f1 (ppm)

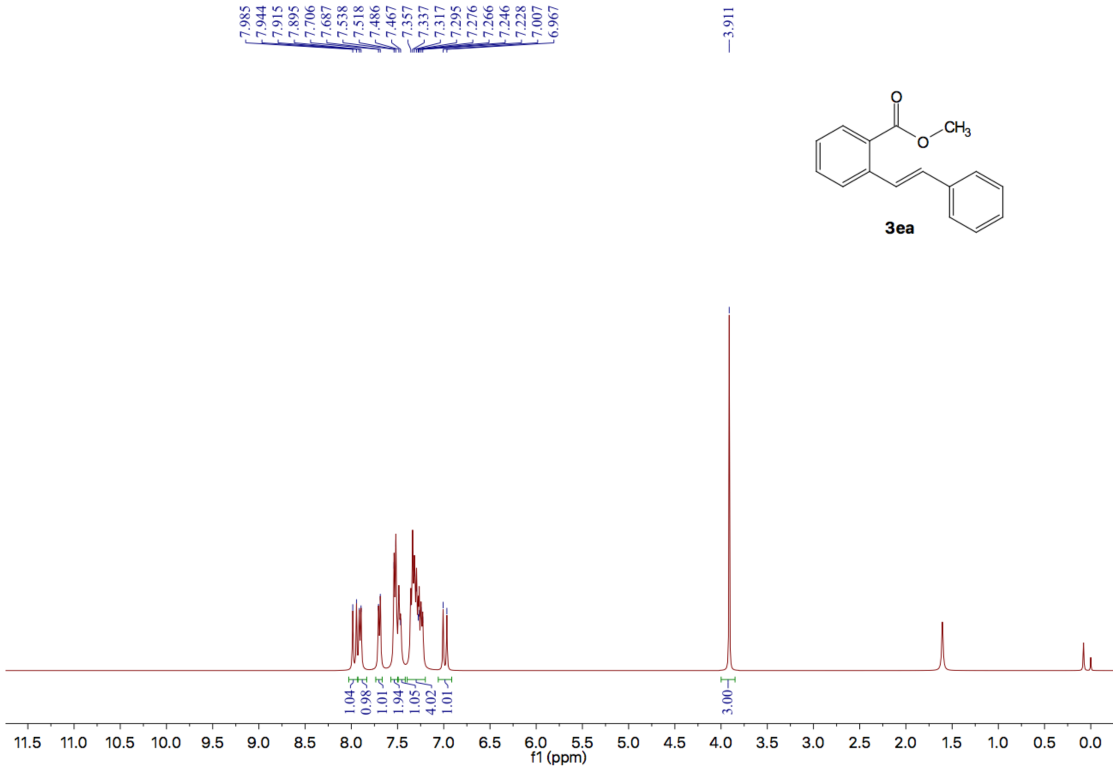
7.985
7.944
7.915
7.895
7.706
7.687
7.538
7.518
7.486
7.467
7.357
7.337
7.317
7.295
7.276
7.266
7.246
7.228
7.007
6.967
3.911
3ea
1.04
0.98
1.01
1.94
1.05
4.02
1.01
3.00
f1 (ppm)

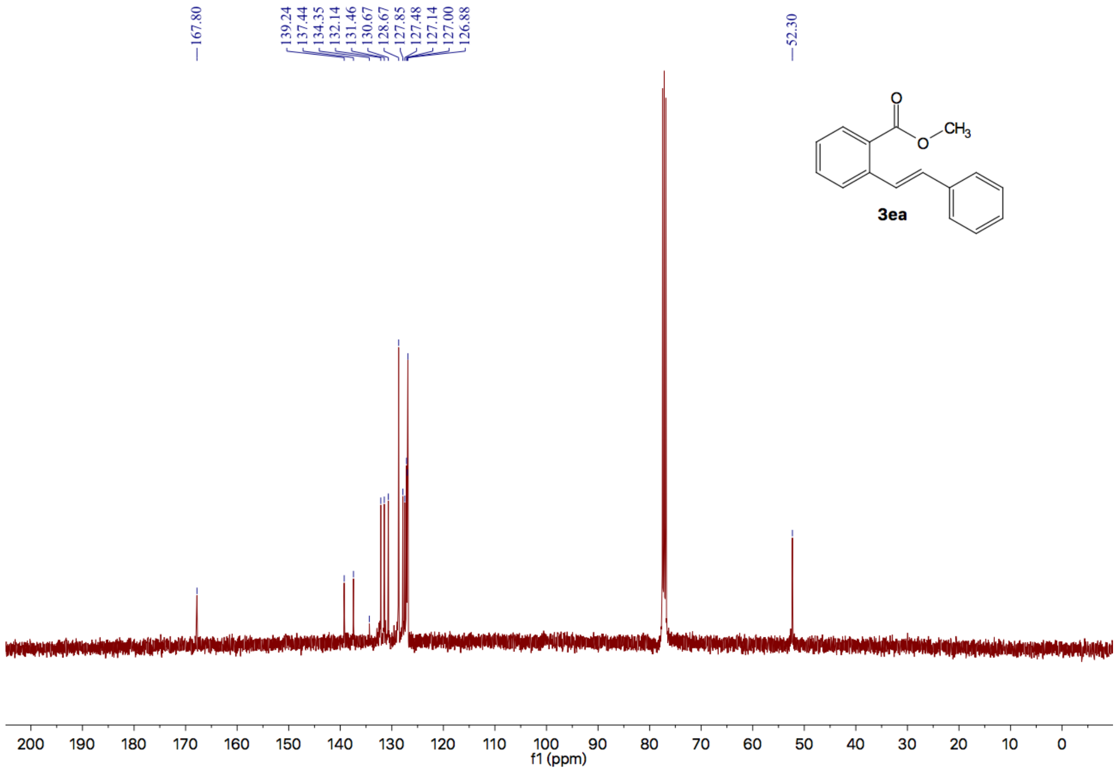
167.80
139.24
137.44
134.35
132.14
131.46
130.67
128.67
127.85
127.48
127.14
127.00
126.88
52.30
3ea
f1 (ppm)

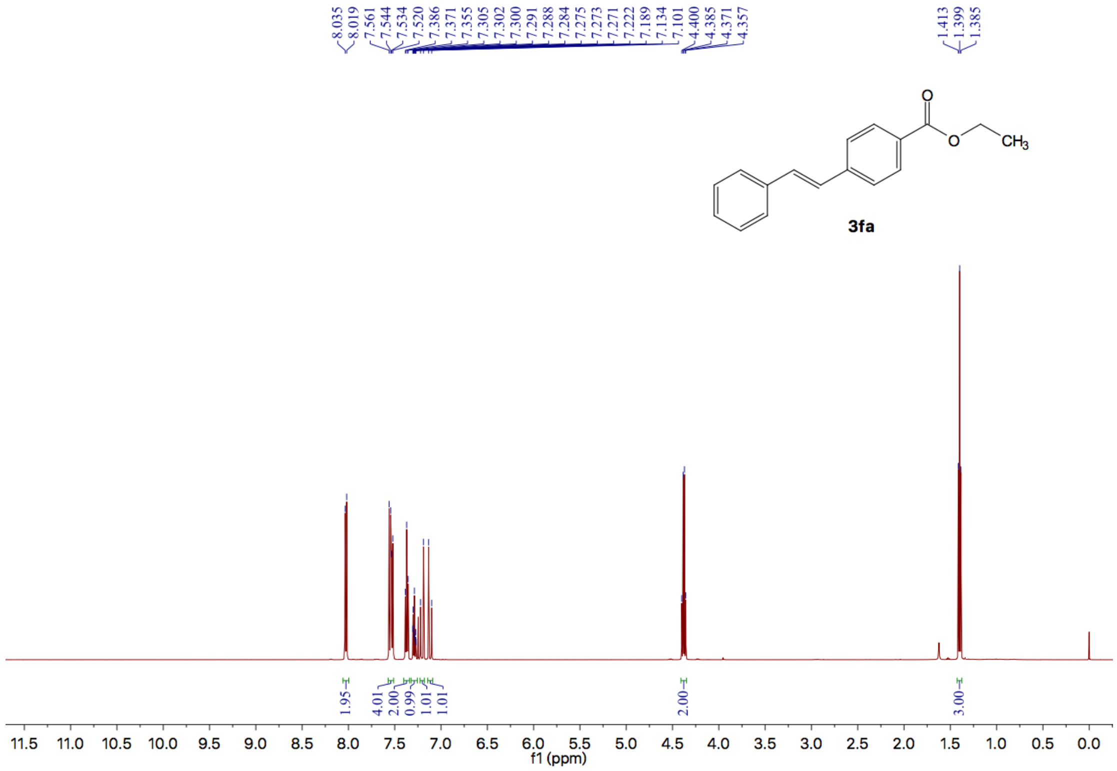

8.035
8.019
7.561
7.544
7.534
7.520
7.386
7.371
7.355
7.305
7.302
7.300
7.291
7.288
7.284
7.275
7.273
7.271
7.222
7.189
7.134
7.101
4.400
4.385
4.371
4.357
1.413
1.399
1.385
O
O
CH3
3fa
1.95
4.01
2.00
0.99
1.01
1.01
2.00
3.00
11.5 11.0 10.5 10.0 9.5 9.0 8.5 8.0 7.5 7.0 6.5 6.0 5.5 5.0 4.5 4.0 3.5 3.0 2.5 2.0 1.5 1.0 0.5 0.0
f1 (ppm)

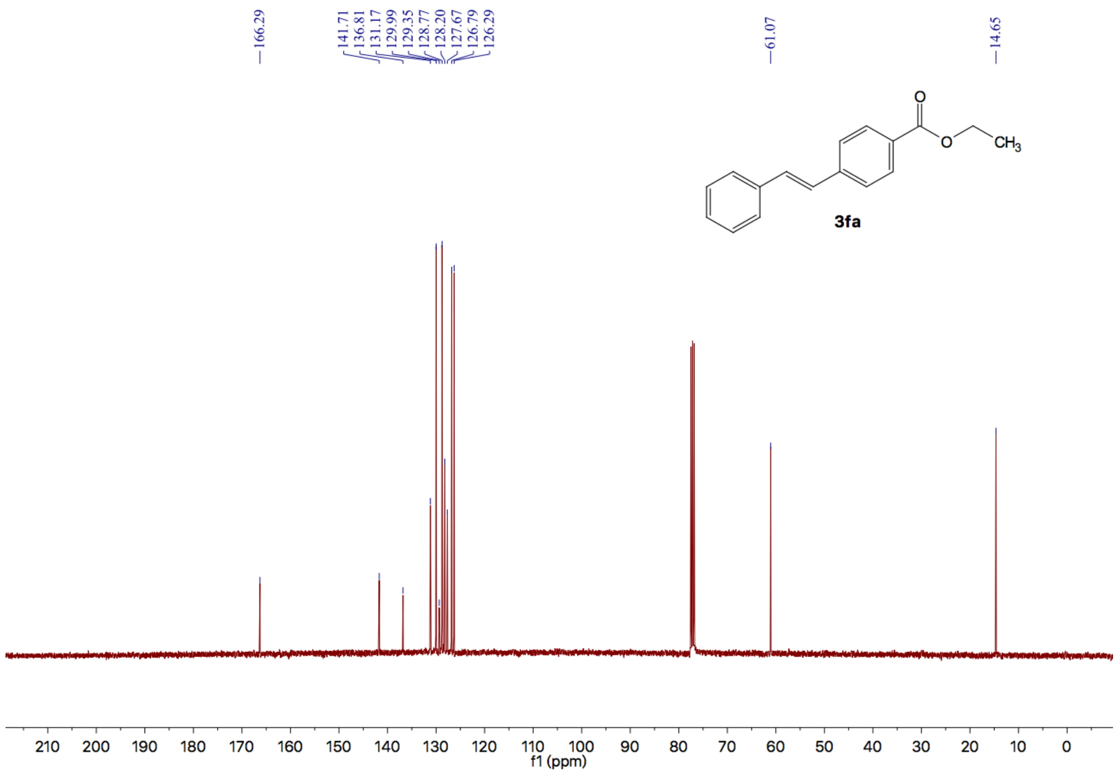
3fa
f1 (ppm)

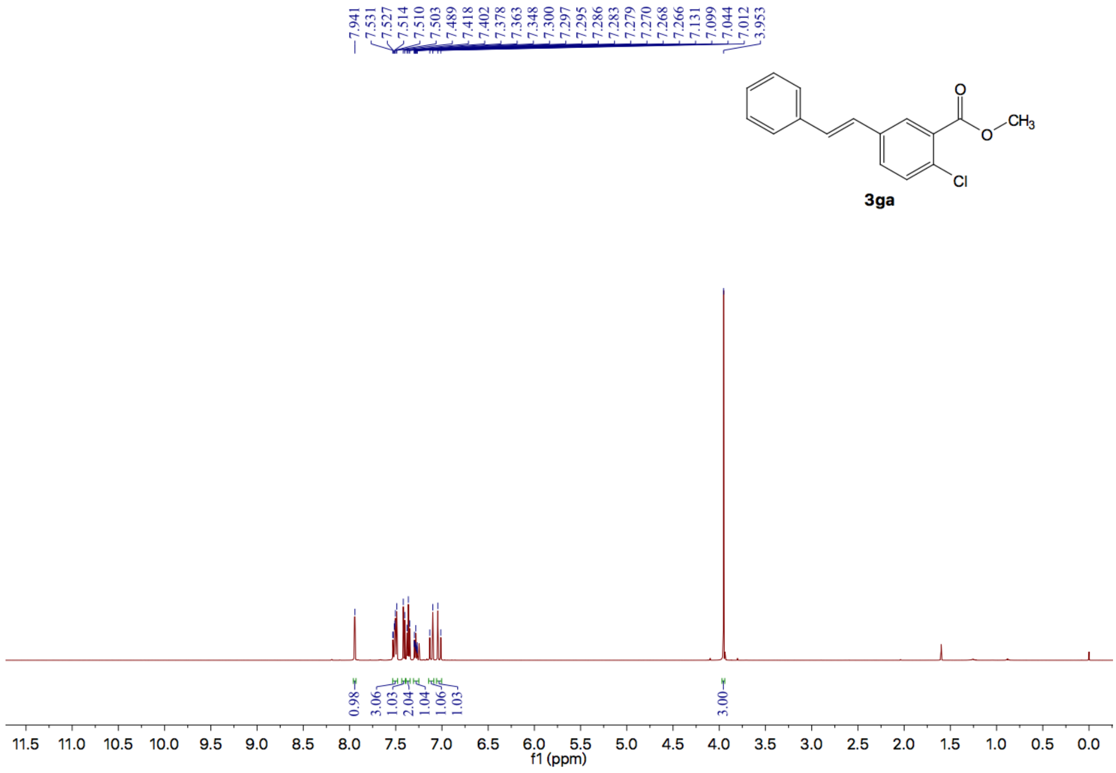
3ga
f1 (ppm)

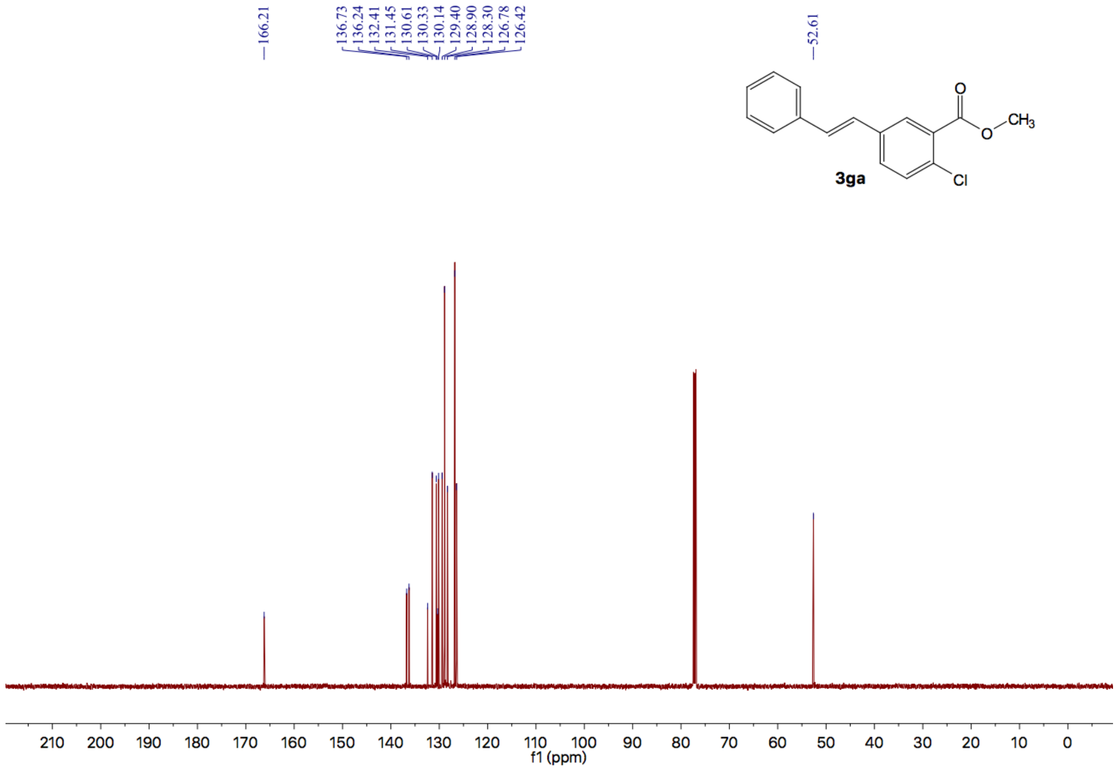

166.21
136.73
136.24
132.41
131.45
130.61
130.33
130.14
129.40
128.90
128.30
126.78
126.42
52.61
O
CH3
Cl
3ga
210 200 190 180 170 160 150 140 130 120 110 100 90 80 70 60 50 40 30 20 10 0
f1 (ppm)

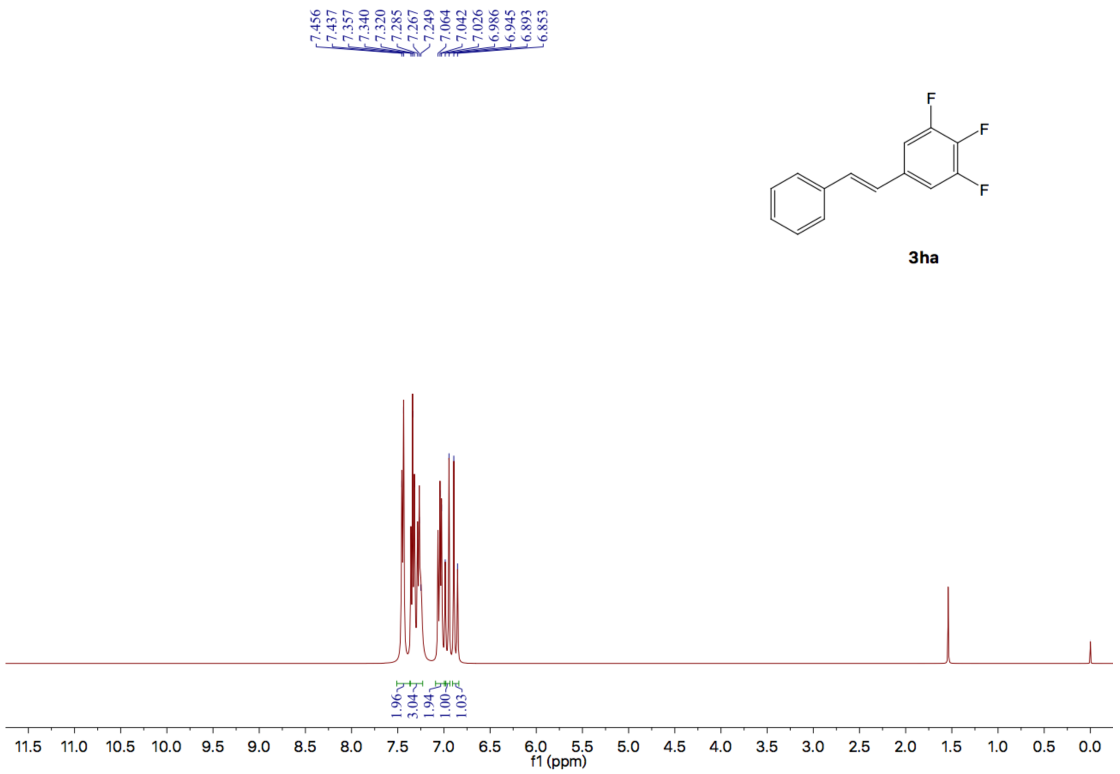
7.456
7.437
7.357
7.340
7.320
7.285
7.267
7.249
7.064
7.042
7.026
6.986
6.945
6.893
6.853
F
F
F
3ha
1.96
3.04
1.94
1.00
1.03
11.5 11.0 10.5 10.0 9.5 9.0 8.5 8.0 7.5 7.0 6.5 6.0 5.5 5.0 4.5 4.0 3.5 3.0 2.5 2.0 1.5 1.0 0.5 0.0
f1 (ppm)

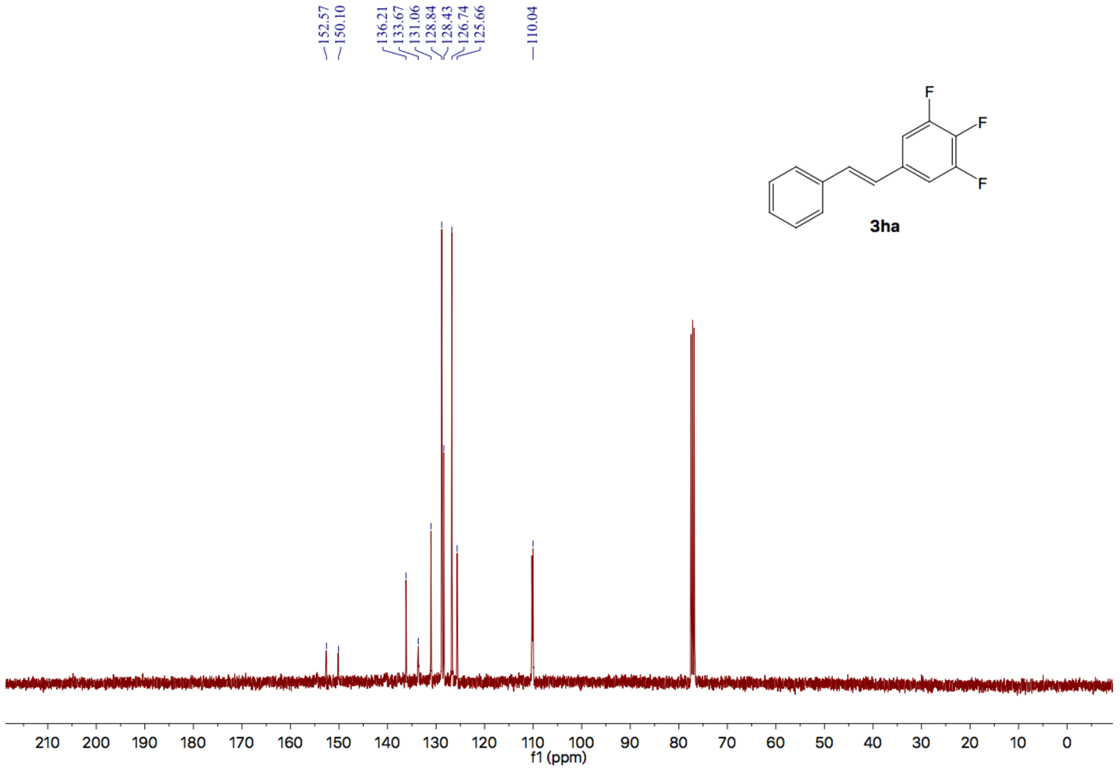
152.57
150.10
136.21
133.67
131.06
128.84
128.43
126.74
125.66
110.04
F
F
F
3ha
210 200 190 180 170 160 150 140 130 120 110 100 90 80 70 60 50 40 30 20 10 0
f1 (ppm)

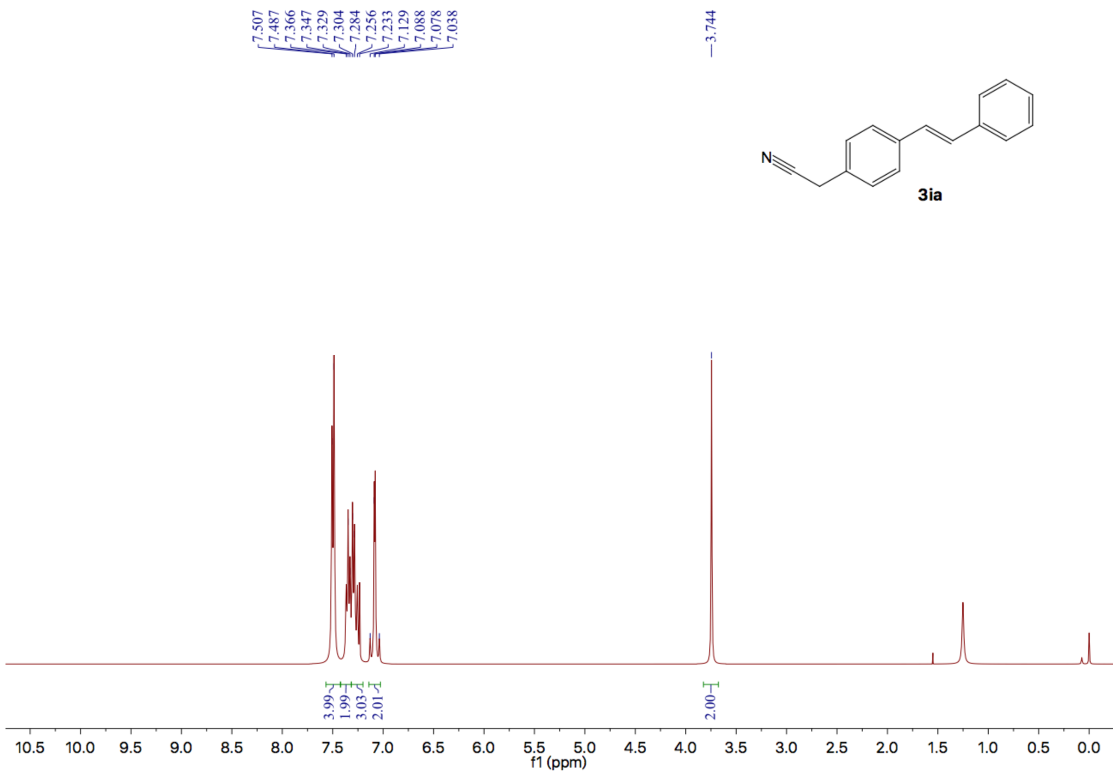
7.507
7.487
7.366
7.347
7.329
7.304
7.284
7.256
7.233
7.129
7.088
7.078
7.038
3.744
3.99
1.99
3.03
2.01
2.00
N
3ia
f1 (ppm)

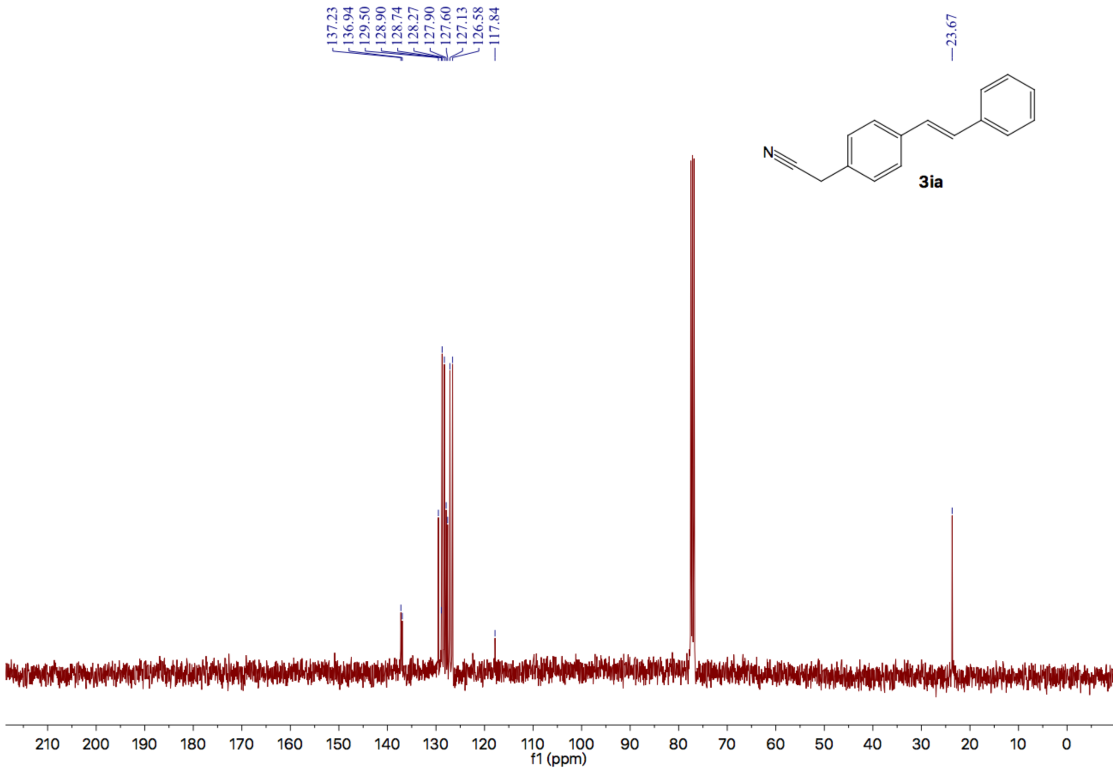
137.23
136.94
129.50
128.90
128.74
128.27
127.90
127.60
127.13
126.58
117.84
23.67
N
3ia
f1 (ppm)

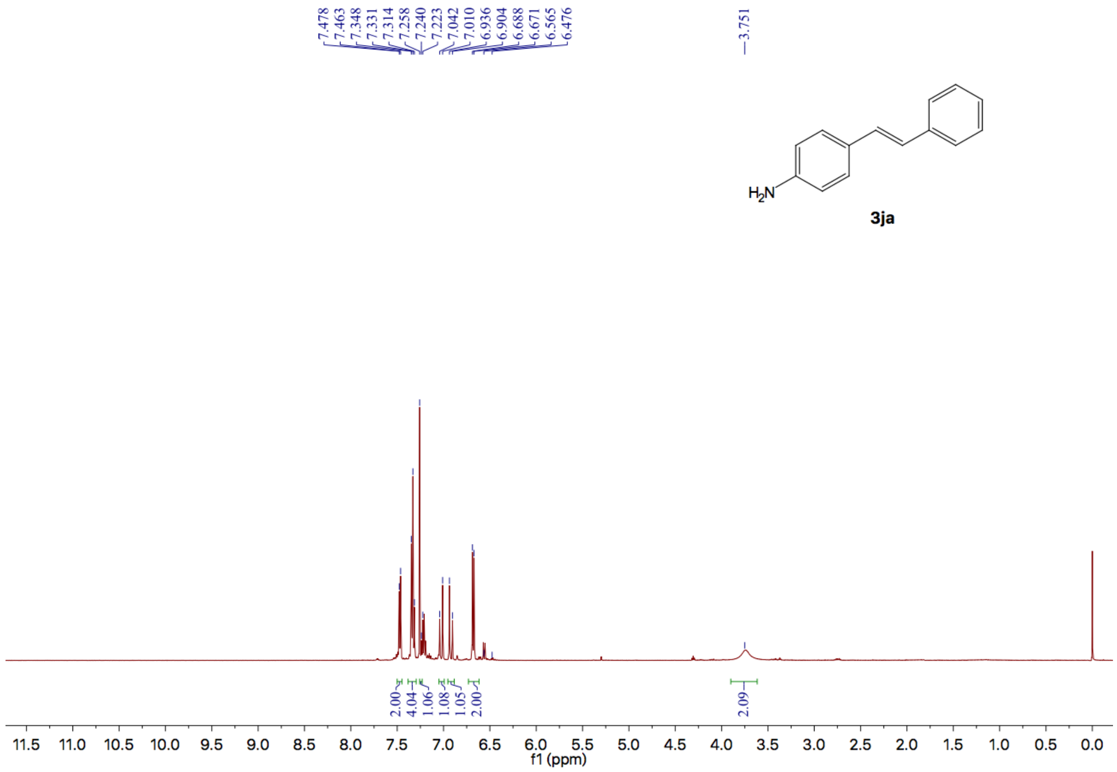
7.478
7.463
7.348
7.331
7.314
7.258
7.240
7.223
7.042
7.010
6.936
6.904
6.688
6.671
6.565
6.476
3.751
H2N
3ja
2.00
4.04
1.06
1.08
1.05
2.00
2.09
11.5 11.0 10.5 10.0 9.5 9.0 8.5 8.0 7.5 7.0 6.5 6.0 5.5 5.0 4.5 4.0 3.5 3.0 2.5 2.0 1.5 1.0 0.5 0.0
f1 (ppm)

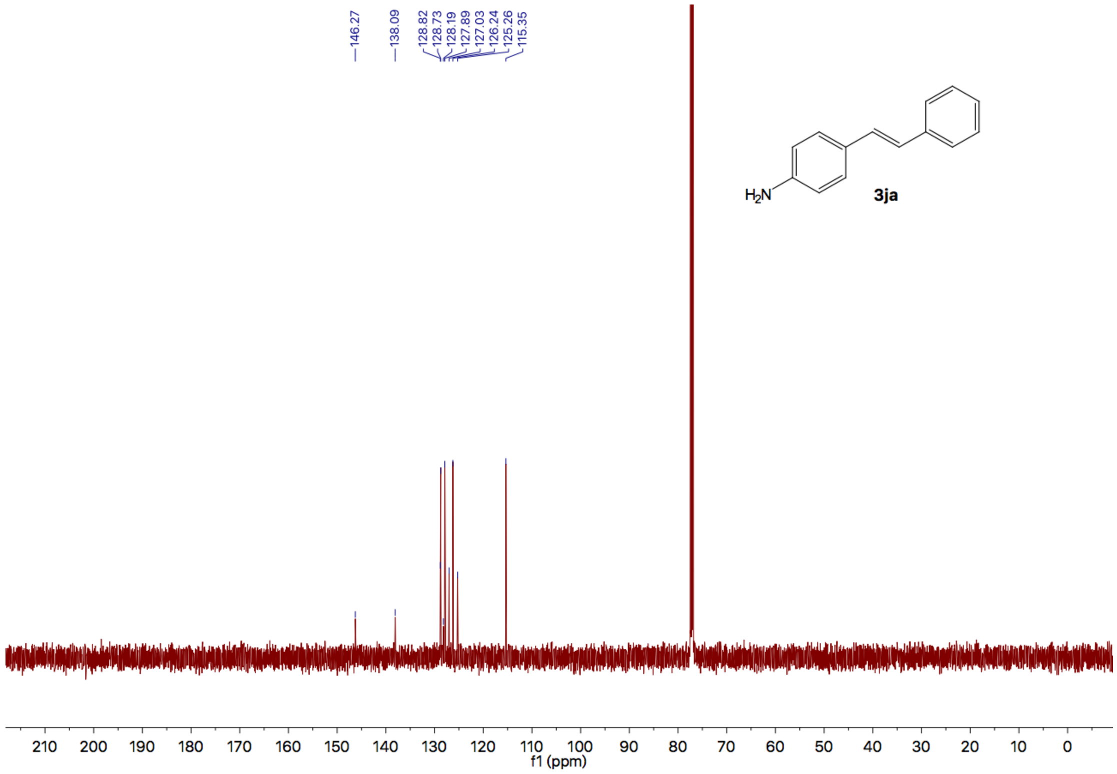

146.27
138.09
128.82
128.73
128.19
127.89
127.03
126.24
125.26
115.35
H2N
3ja
210 200 190 180 170 160 150 140 130 120 110 100 90 80 70 60 50 40 30 20 10 0
f1 (ppm)

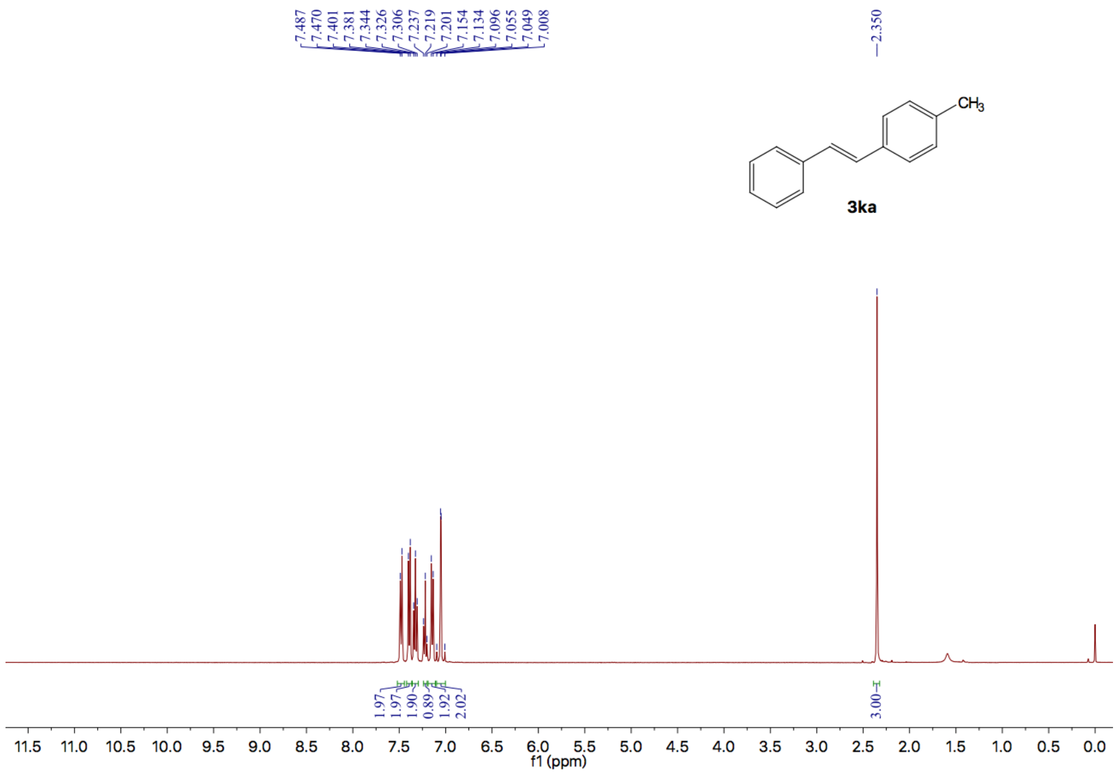
7.487
7.470
7.401
7.381
7.344
7.326
7.306
7.237
7.219
7.201
7.154
7.134
7.096
7.055
7.049
7.008
2.350
CH_3
3ka
1.97
1.97
1.90
0.89
1.92
2.02
3.00
11.5 11.0 10.5 10.0 9.5 9.0 8.5 8.0 7.5 7.0 6.5 6.0 5.5 5.0 4.5 4.0 3.5 3.0 2.5 2.0 1.5 1.0 0.5 0.0
f1 (ppm)

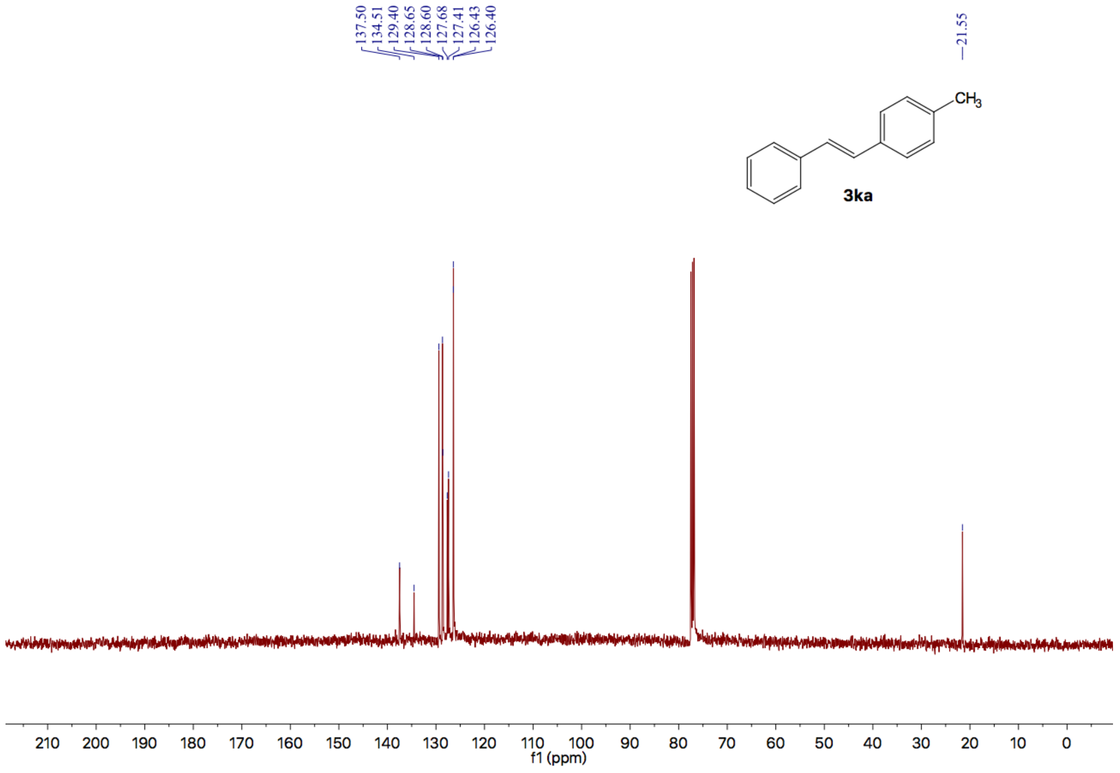
137.50
134.51
129.40
128.65
128.60
127.68
127.41
126.43
126.40
21.55
CH_3
3ka
210 200 190 180 170 160 150 140 130 120 110 100 90 80 70 60 50 40 30 20 10 0
f1 (ppm)

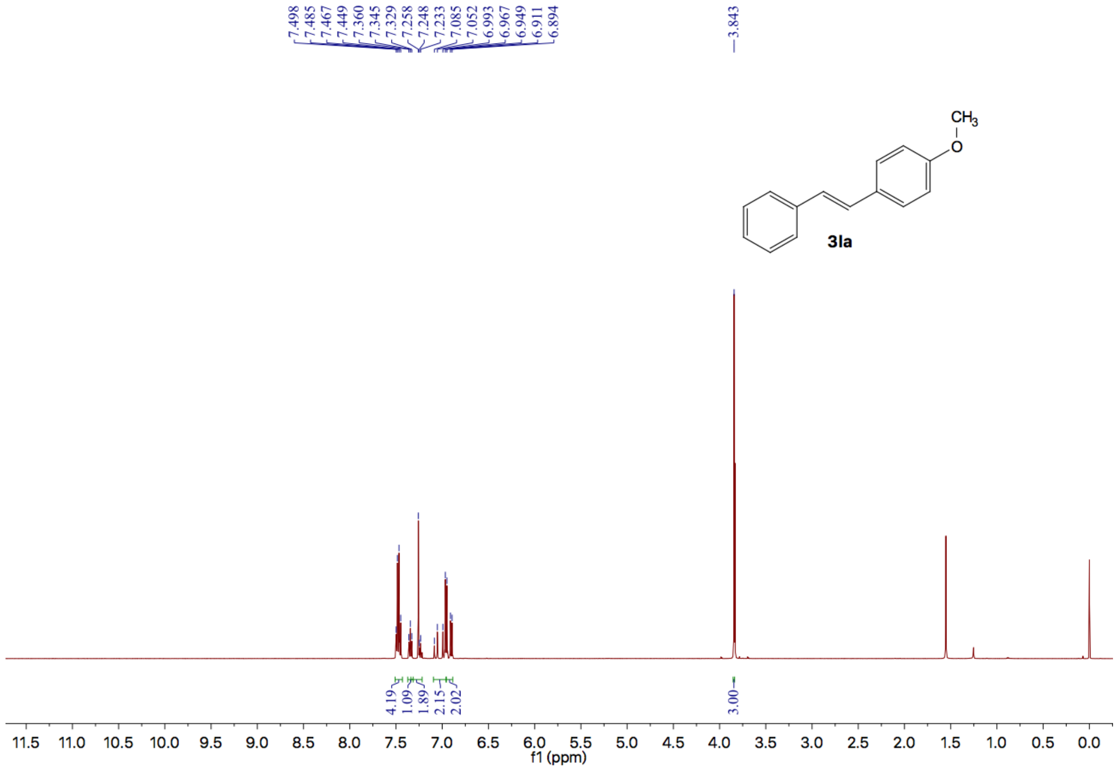
7.498
7.485
7.467
7.449
7.360
7.345
7.329
7.258
7.248
7.233
7.085
7.052
6.993
6.967
6.949
6.911
6.894
3.843
CH3
O
3la
4.19
1.09
1.89
2.15
2.02
3.00
11.5 11.0 10.5 10.0 9.5 9.0 8.5 8.0 7.5 7.0 6.5 6.0 5.5 5.0 4.5 4.0 3.5 3.0 2.5 2.0 1.5 1.0 0.5 0.0
f1 (ppm)

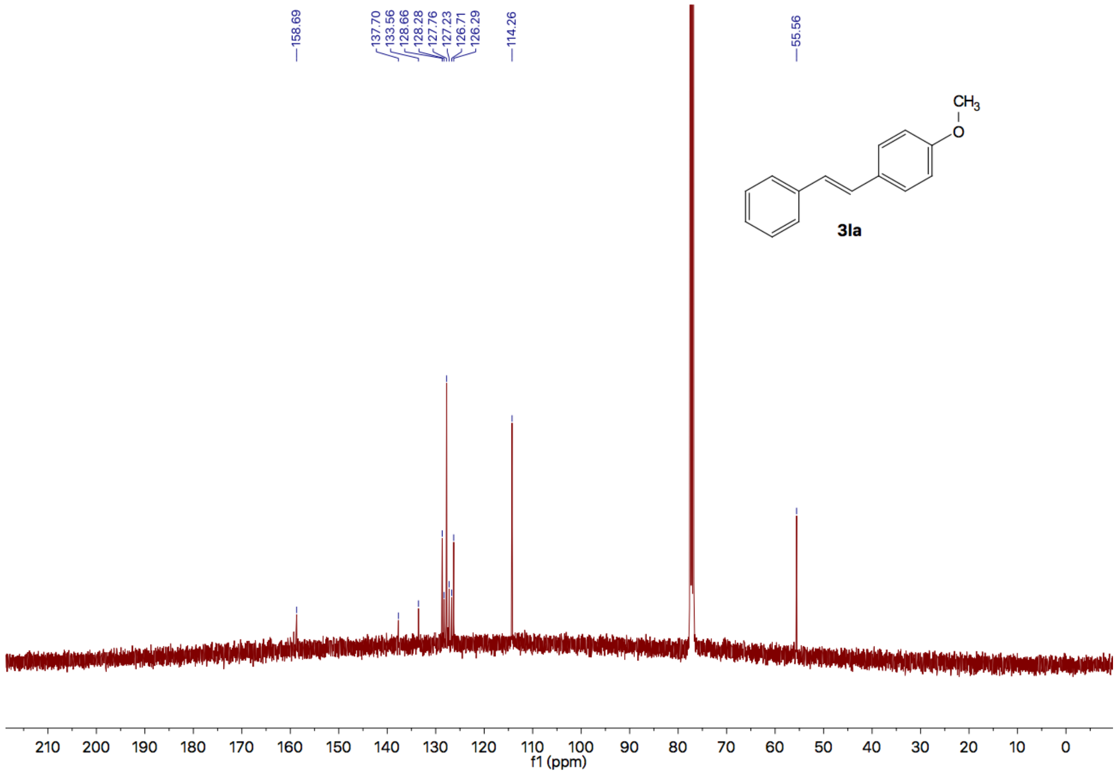
158.69
137.70
133.56
128.66
128.28
127.76
127.23
126.71
126.29
114.26
55.56
CH3
O
3la
210 200 190 180 170 160 150 140 130 120 110 100 90 80 70 60 50 40 30 20 10 0
f1 (ppm)

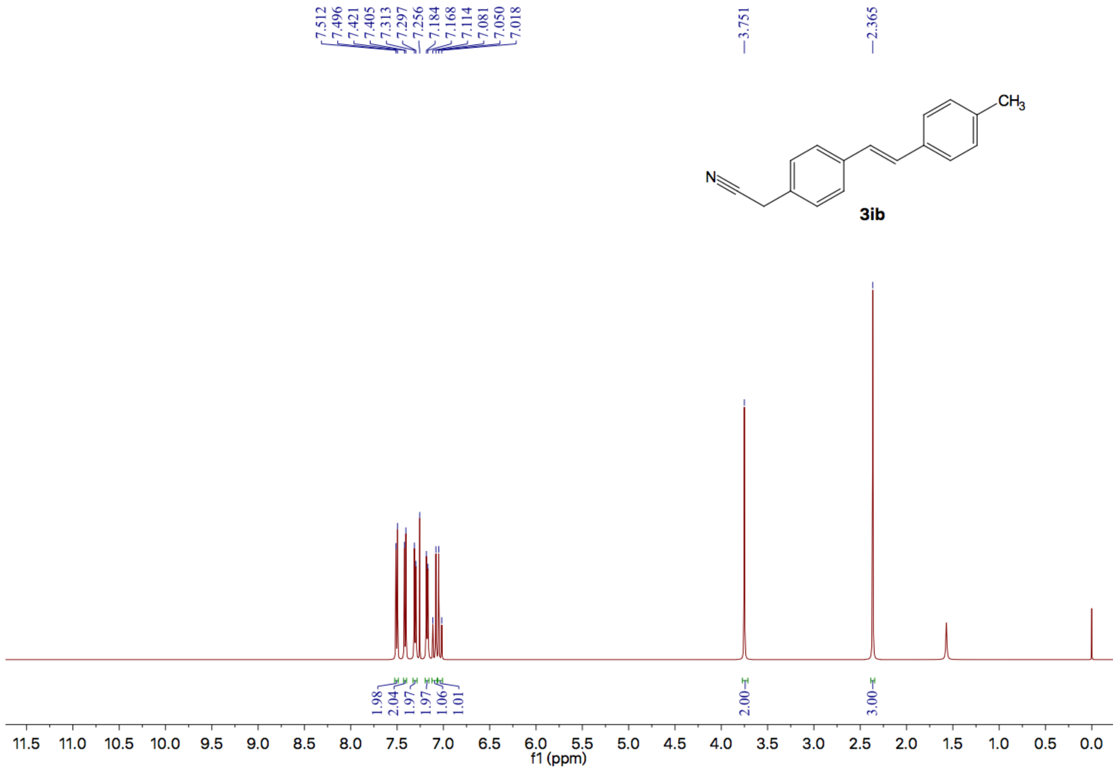
7.512
7.496
7.421
7.405
7.313
7.297
7.256
7.184
7.168
7.114
7.081
7.050
7.018
3.751
2.365
CH3
N
3ib
1.98
2.04
1.97
1.97
1.06
1.01
2.00
3.00
11.5 11.0 10.5 10.0 9.5 9.0 8.5 8.0 7.5 7.0 6.5 6.0 5.5 5.0 4.5 4.0 3.5 3.0 2.5 2.0 1.5 1.0 0.5 0.0
f1 (ppm)

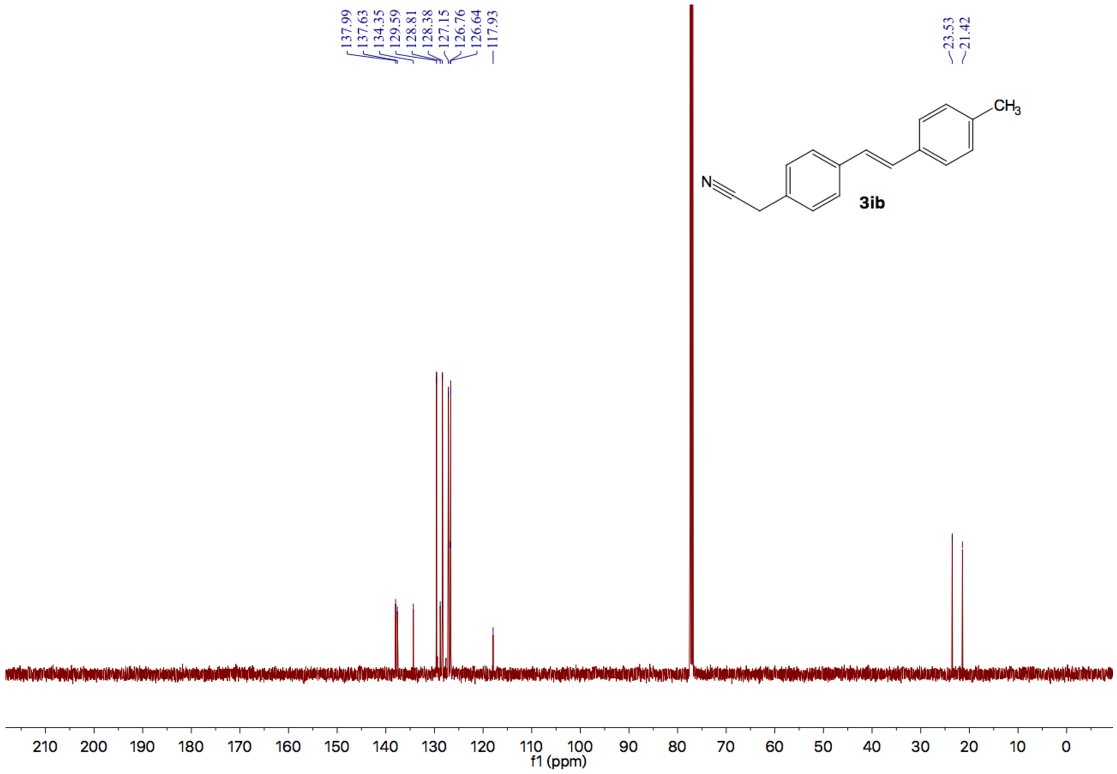
137.99
137.63
134.35
129.59
128.81
128.38
127.15
126.76
126.64
117.93
23.53
21.42
CH3
N
3ib
210 200 190 180 170 160 150 140 130 120 110 100 90 80 70 60 50 40 30 20 10 0
f1 (ppm)

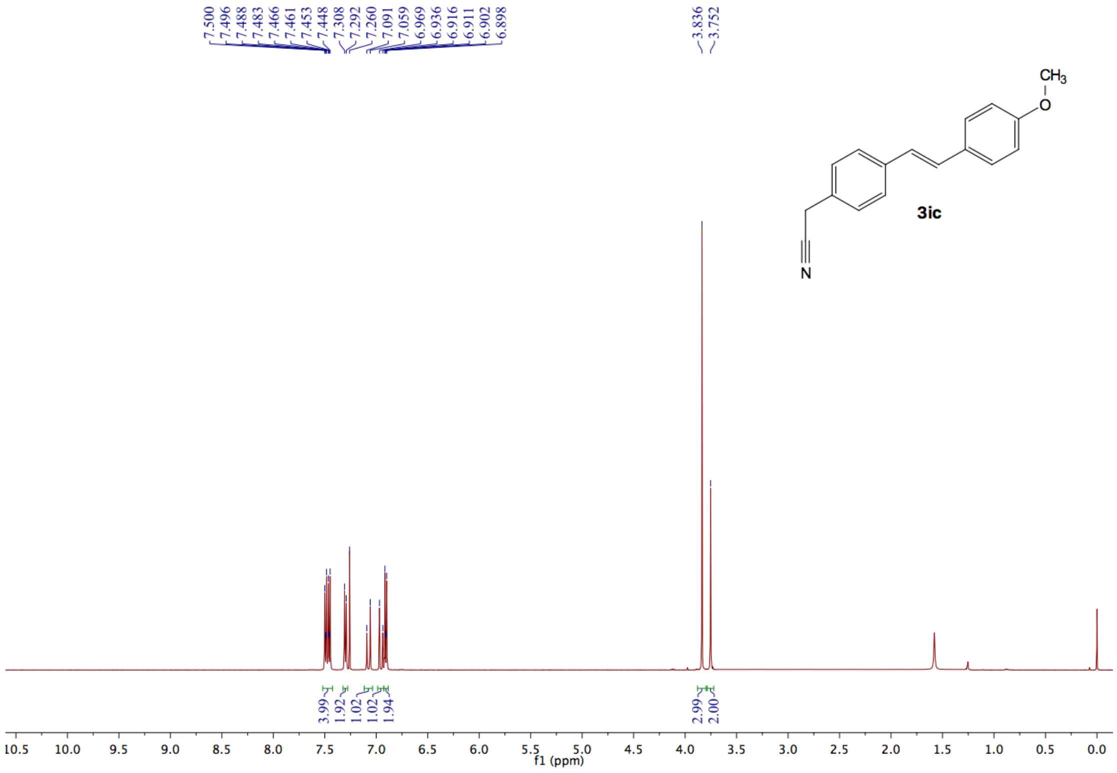
7.500
7.496
7.488
7.483
7.466
7.461
7.453
7.448
7.308
7.292
7.260
7.091
7.059
6.969
6.936
6.916
6.911
6.902
6.898
3.836
3.752
CH3
O
N
3ic
3.99
1.92
1.02
1.02
1.94
2.99
2.00
10.5 10.0 9.5 9.0 8.5 8.0 7.5 7.0 6.5 6.0 5.5 5.0 4.5 4.0 3.5 3.0 2.5 2.0 1.5 1.0 0.5 0.0
f1 (ppm)

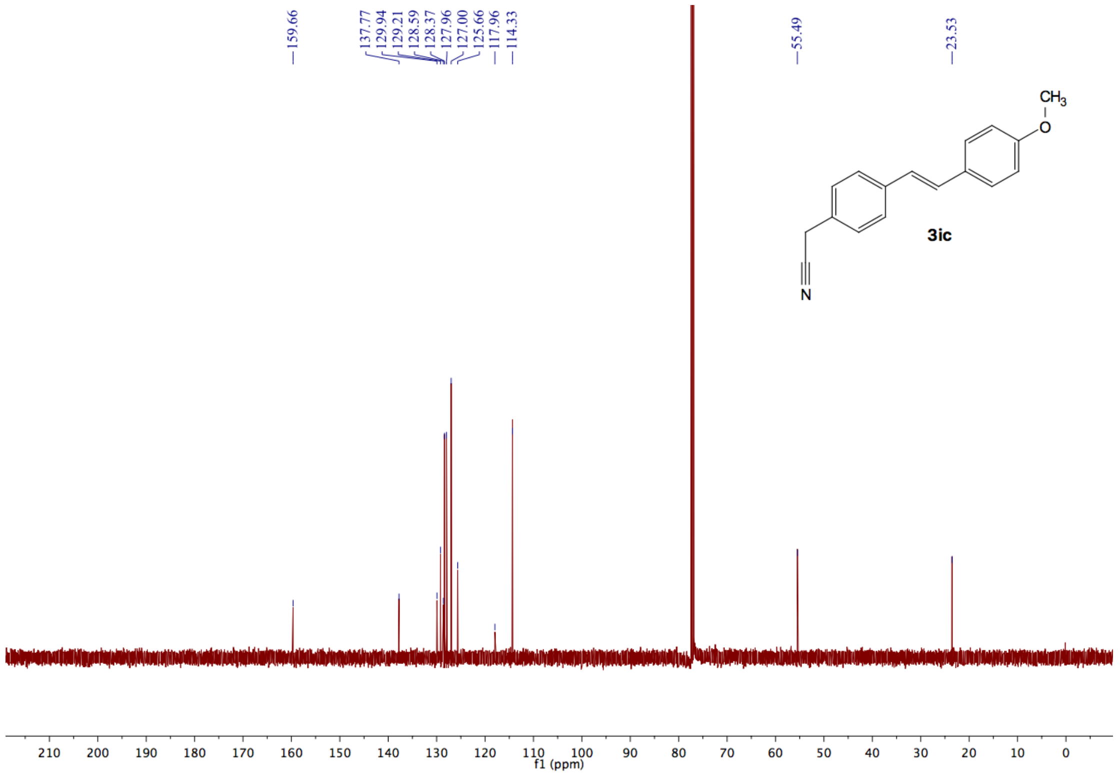

159.66
137.77
129.94
129.21
128.59
128.37
127.96
127.00
125.66
117.96
114.33
55.49
23.53
CH3
O
N
3ic
210
200
190
180
170
160
150
140
130
120
110
100
90
80
70
60
50
40
30
20
10
0
f1 (ppm)

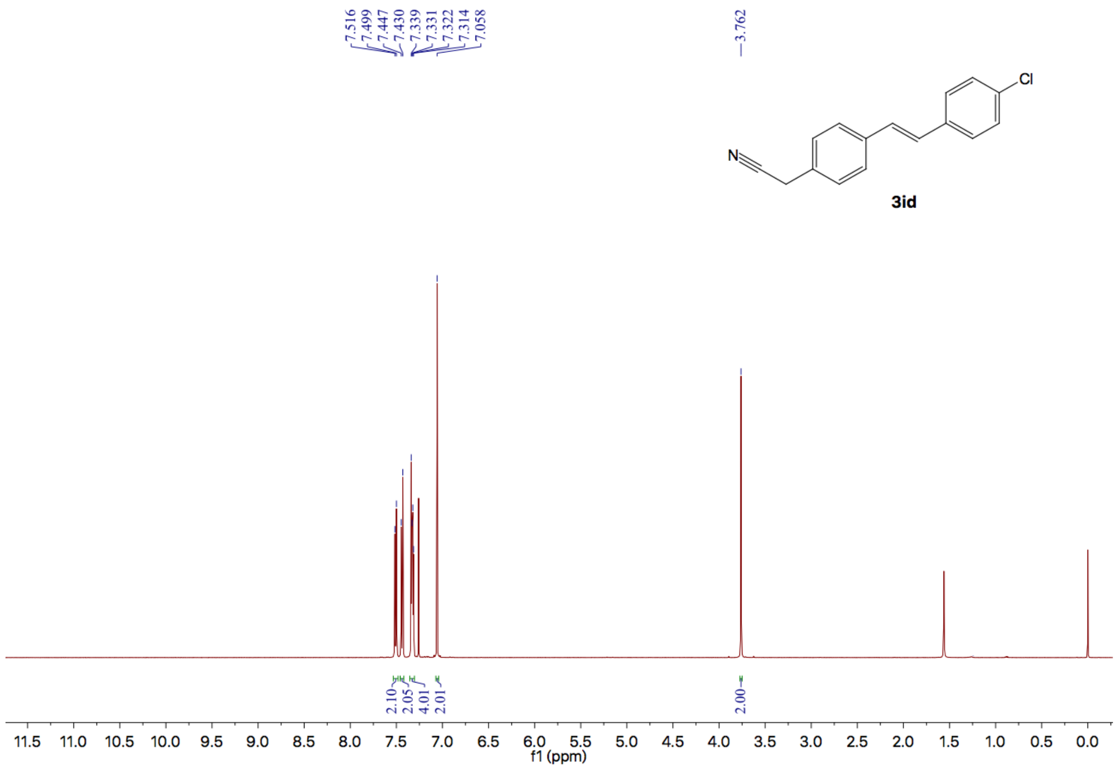
7.516
7.499
7.447
7.430
7.339
7.331
7.322
7.314
7.058
3.762
N
Cl
3id
2.10
2.05
4.01
2.01
2.00
11.5 11.0 10.5 10.0 9.5 9.0 8.5 8.0 7.5 7.0 6.5 6.0 5.5 5.0 4.5 4.0 3.5 3.0 2.5 2.0 1.5 1.0 0.5 0.0
f1 (ppm)

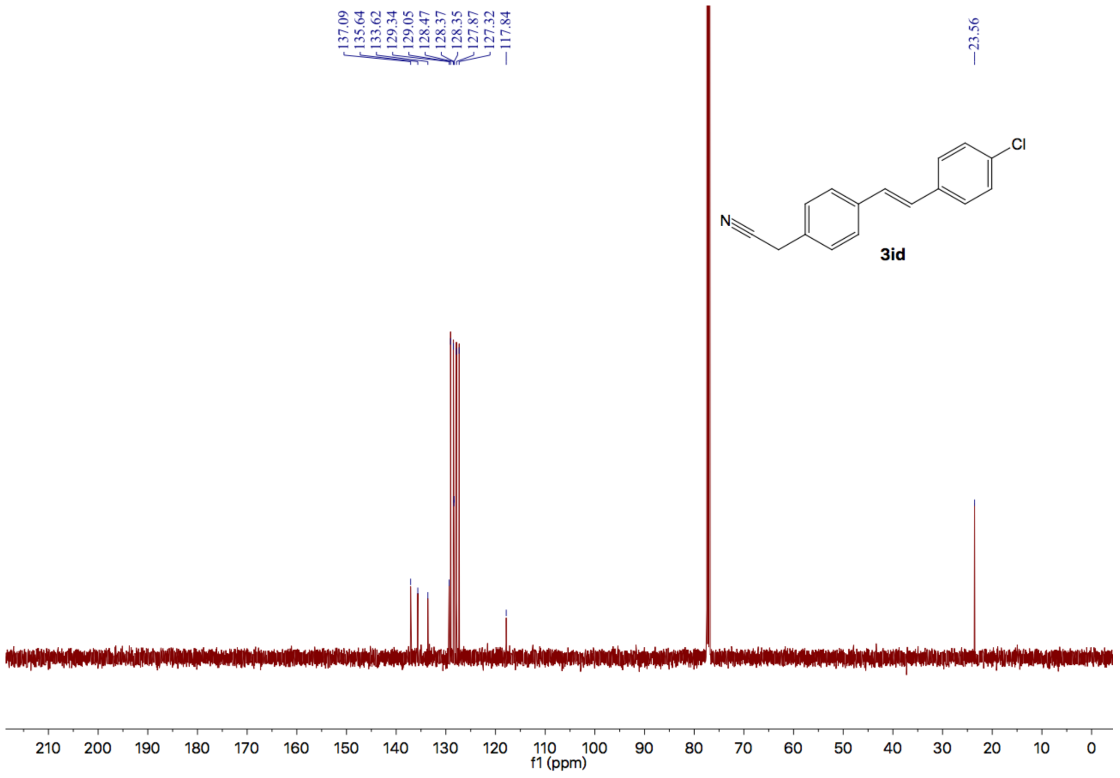
137.09
135.64
133.62
129.34
129.05
128.47
128.37
128.35
127.87
127.32
117.84
23.56
N
Cl
3id
210 200 190 180 170 160 150 140 130 120 110 100 90 80 70 60 50 40 30 20 10 0
f1 (ppm)

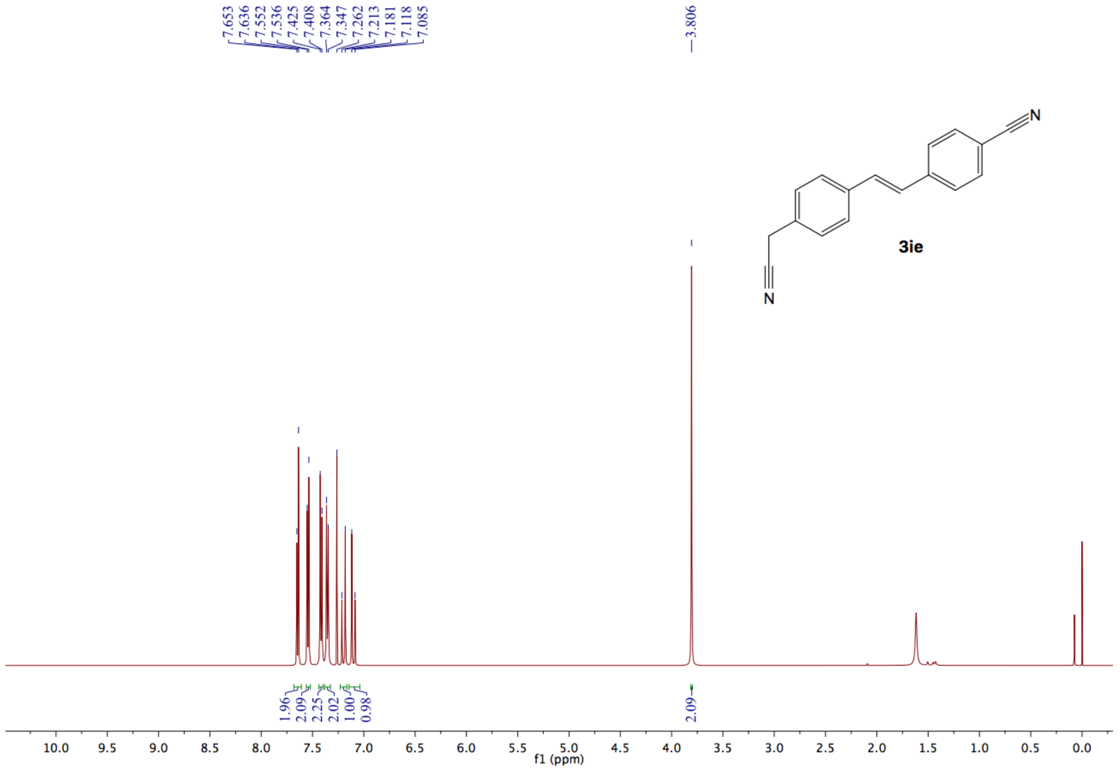
7.653
7.636
7.552
7.536
7.425
7.408
7.364
7.347
7.262
7.213
7.181
7.118
7.085
3.806
N
3ie
N
1.96
2.09
2.25
2.02
1.00
0.98
2.09
10.0
9.5
9.0
8.5
8.0
7.5
7.0
6.5
6.0
5.5
5.0
4.5
4.0
3.5
3.0
2.5
2.0
1.5
1.0
0.5
0.0
f1 (ppm)

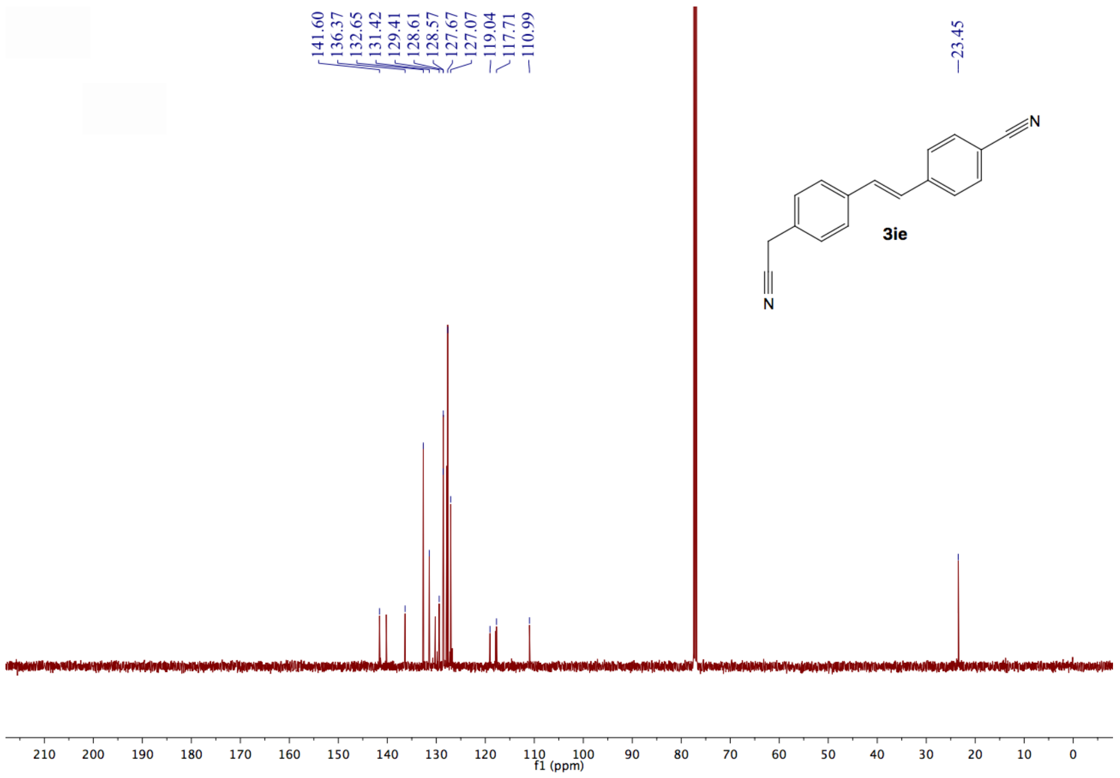
141.60
136.37
132.65
131.42
129.41
128.61
128.57
127.67
127.07
119.04
117.71
110.99
23.45
N
3ie
N
210
200
190
180
170
160
150
140
130
120
110
100
90
80
70
60
50
40
30
20
10
0
f1 (ppm)

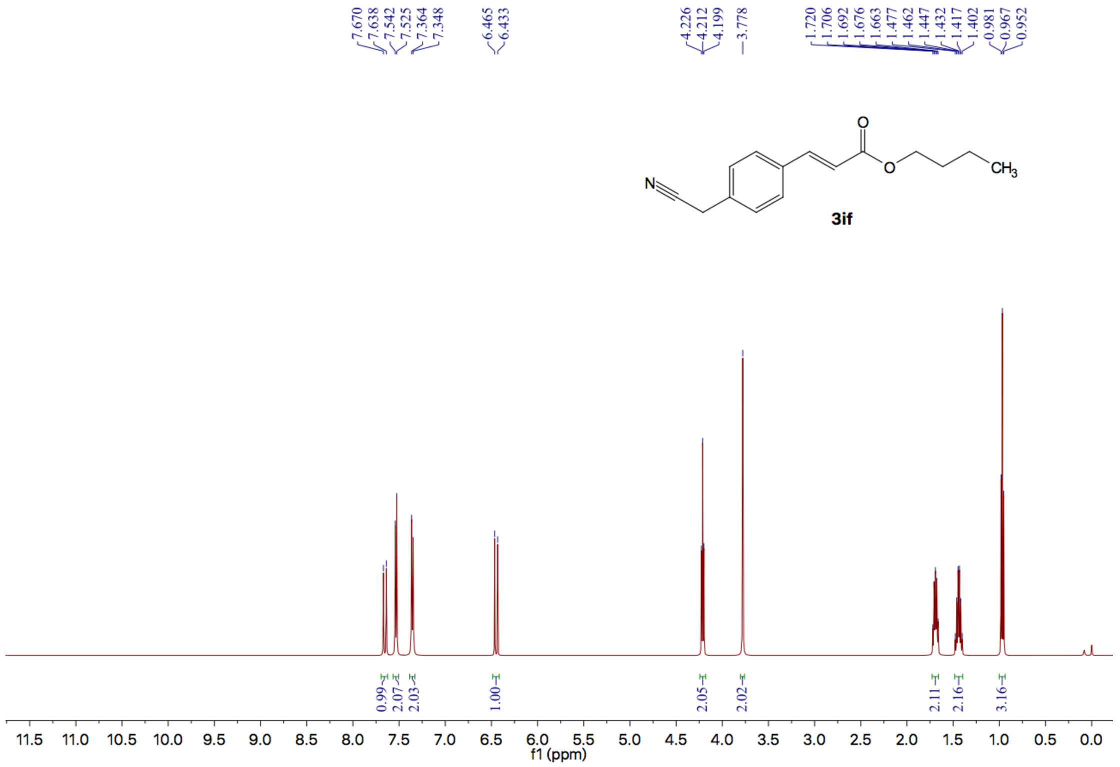
7.670
7.638
7.542
7.525
7.364
7.348
6.465
6.433
4.226
4.212
4.199
3.778
1.720
1.706
1.692
1.676
1.663
1.477
1.462
1.447
1.432
1.417
1.402
0.981
0.967
0.952
O
N
O
CH3
3if
0.99
2.07
2.03
1.00
2.05
2.02
2.11
2.16
3.16
11.5 11.0 10.5 10.0 9.5 9.0 8.5 8.0 7.5 7.0 6.5 6.0 5.5 5.0 4.5 4.0 3.5 3.0 2.5 2.0 1.5 1.0 0.5 0.0
f1 (ppm)

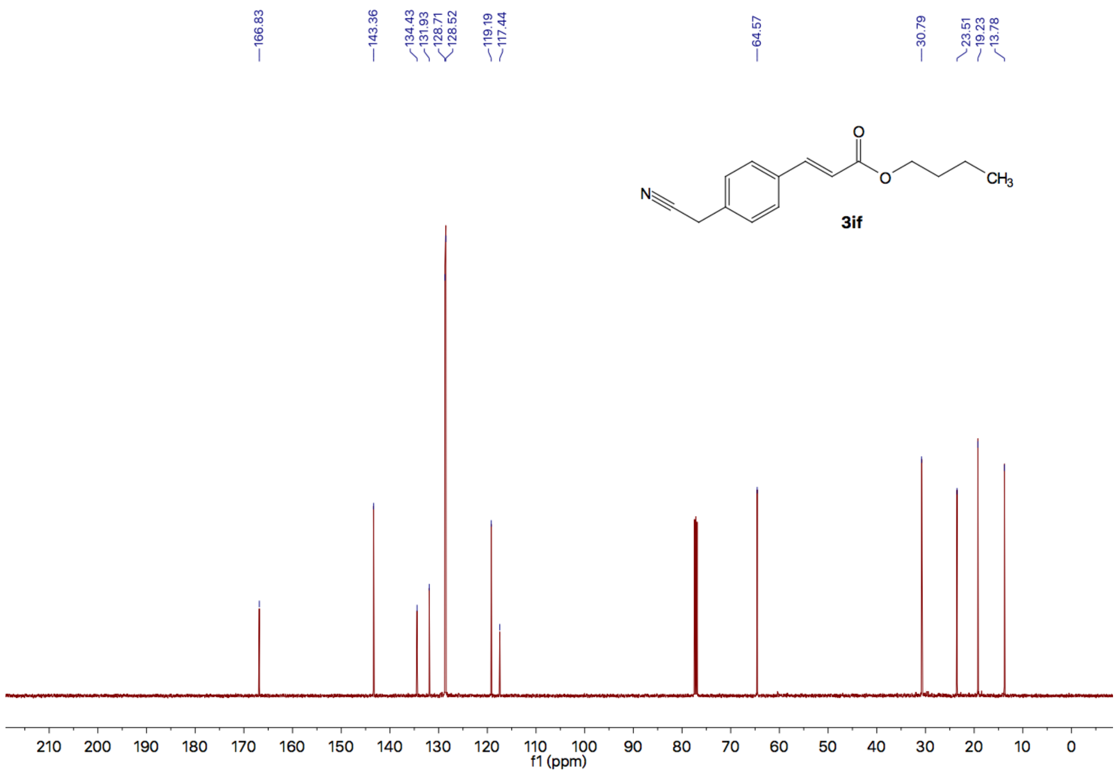
166.83
143.36
134.43
131.93
128.71
128.52
119.19
117.44
64.57
30.79
23.51
19.23
13.78
N
O
O
CH3
3if
210
200
190
180
170
160
150
140
130
120
110
100
90
80
70
60
50
40
30
20
10
0
f1 (ppm)

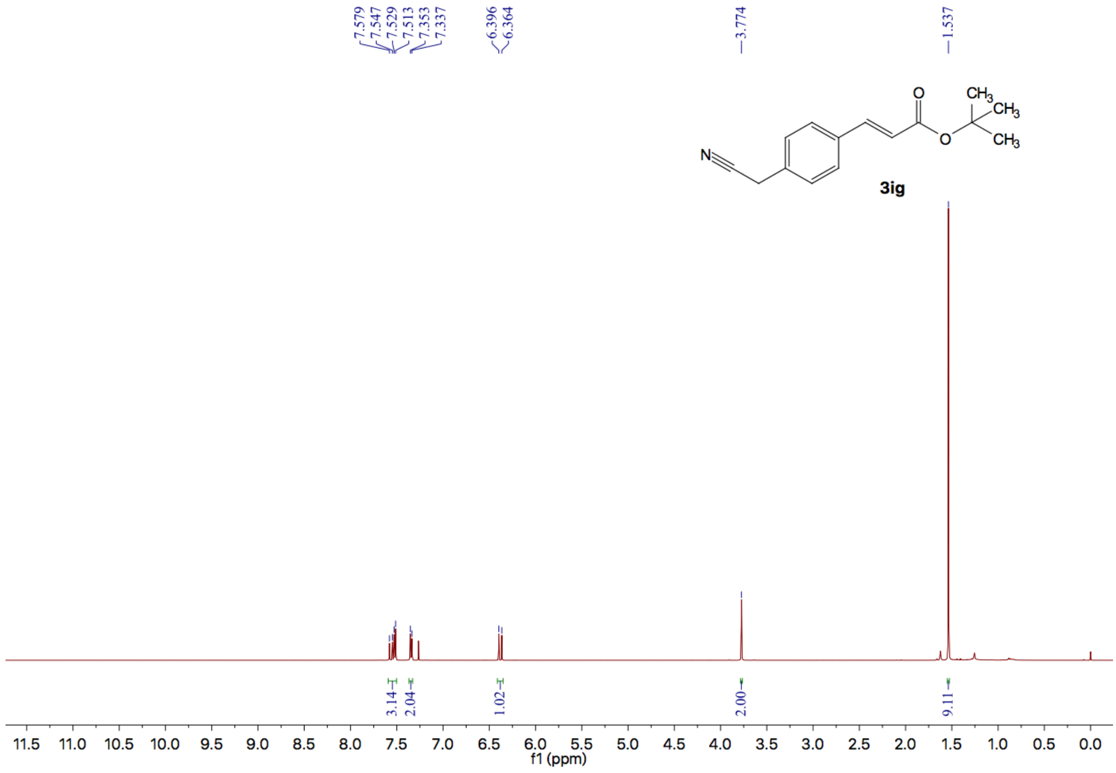
7.579
7.547
7.529
7.513
7.353
7.337
6.396
6.364
3.774
1.537
N
O
O
CH3
CH3
CH3
3ig
3.14
2.04
1.02
2.00
9.11
11.5
11.0
10.5
10.0
9.5
9.0
8.5
8.0
7.5
7.0
6.5
6.0
5.5
5.0
4.5
4.0
3.5
3.0
2.5
2.0
1.5
1.0
0.5
0.0
f1 (ppm)

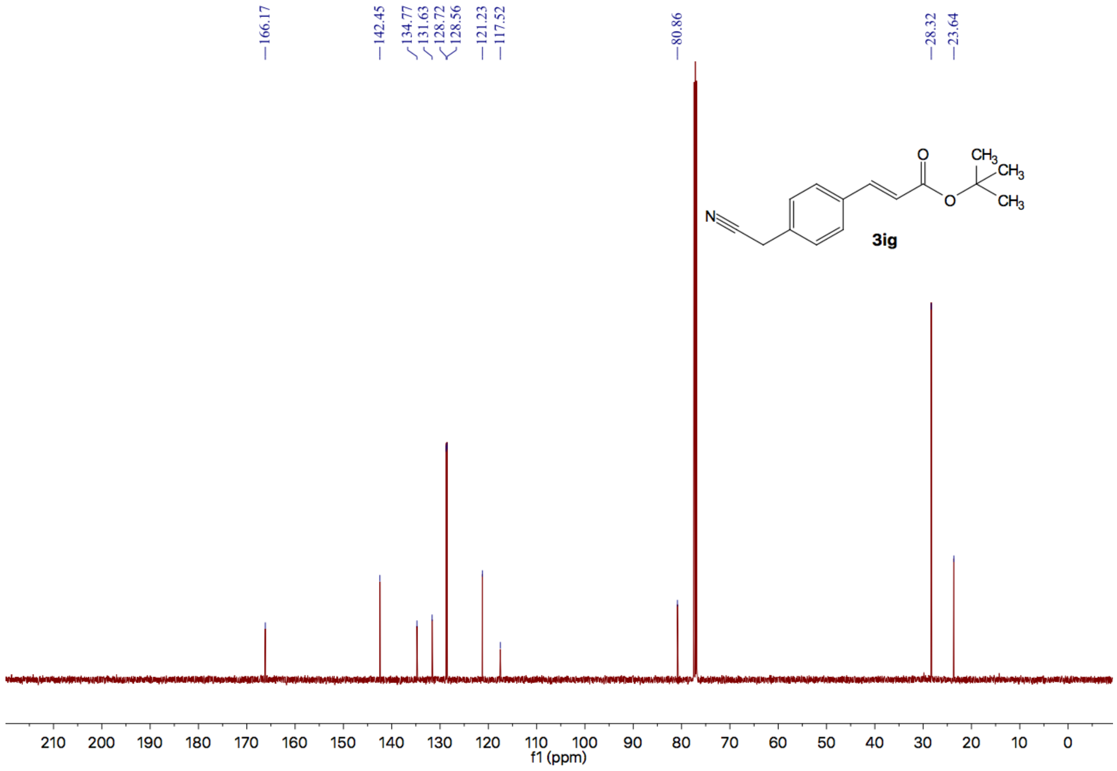
166.17
142.45
134.77
131.63
128.72
128.56
121.23
117.52
80.86
28.32
23.64
N
O
O
CH3
CH3
CH3
3ig
210
200
190
180
170
160
150
140
130
120
110
100
90
80
70
60
50
40
30
20
10
0
f1 (ppm)

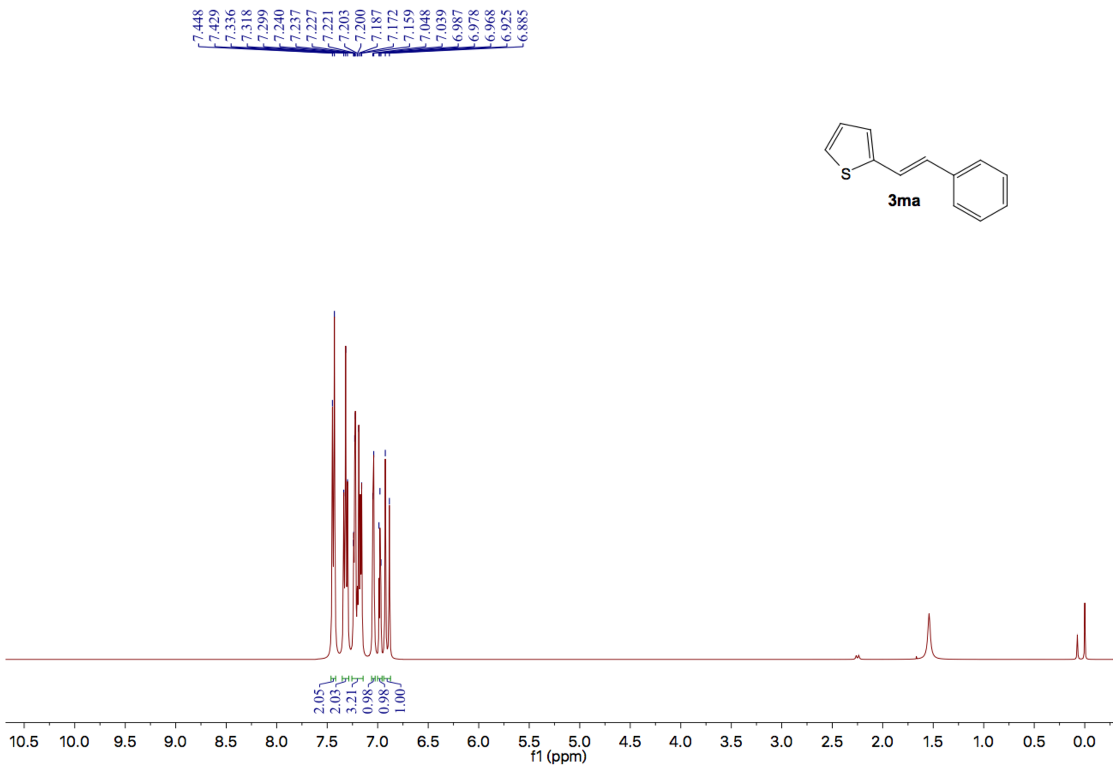
7.448
7.429
7.336
7.318
7.299
7.240
7.237
7.227
7.221
7.203
7.200
7.187
7.172
7.159
7.048
7.039
6.987
6.978
6.968
6.925
6.885
3ma
2.05
2.03
3.21
0.98
0.98
1.00
f1 (ppm)

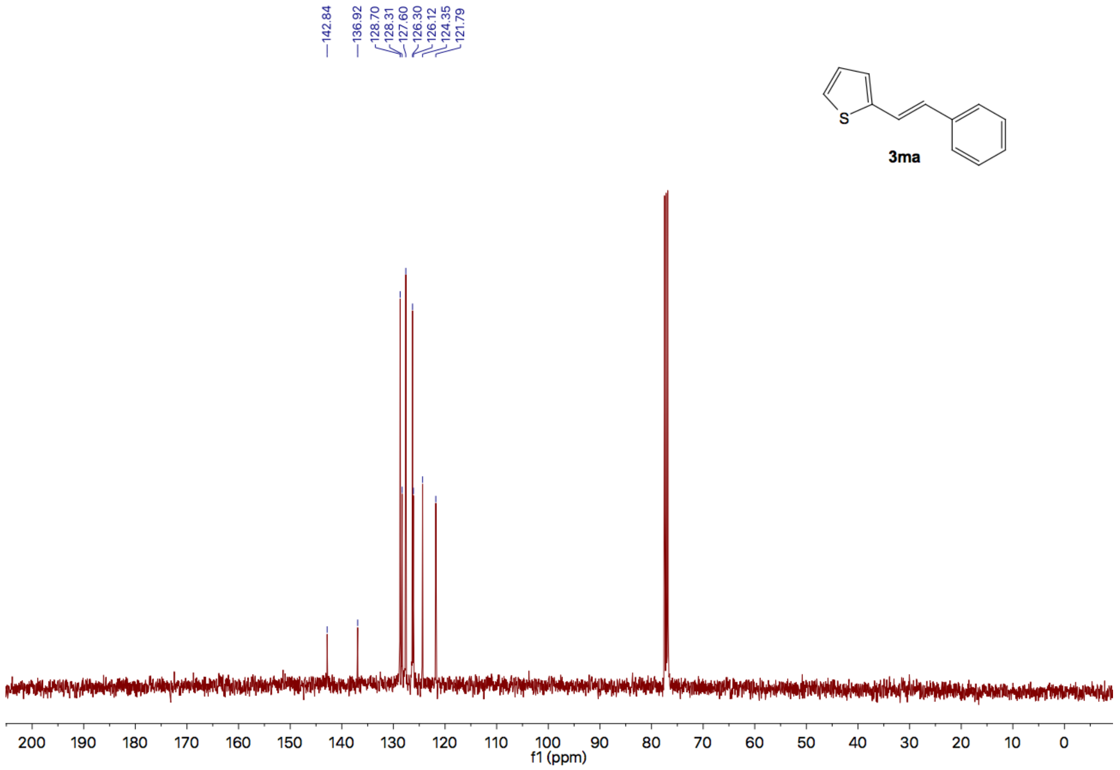
142.84
136.92
128.70
128.31
127.60
126.30
126.12
124.35
121.79
3ma
f1 (ppm)

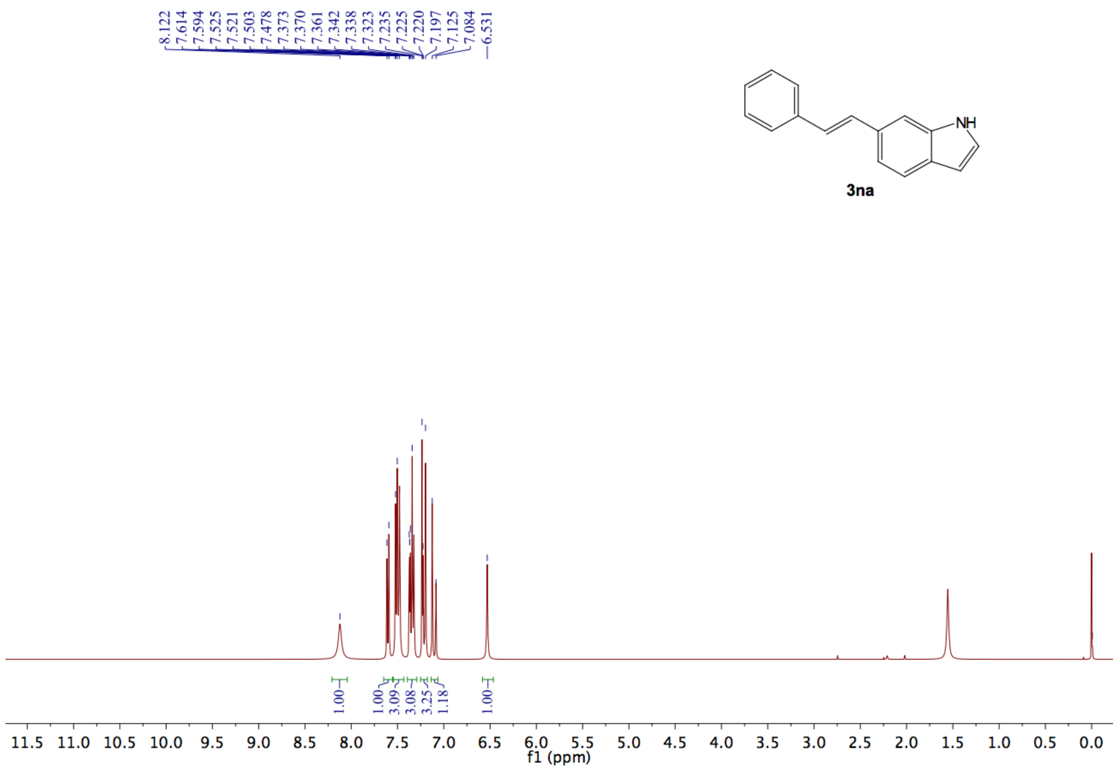
8.122
7.614
7.594
7.525
7.521
7.503
7.478
7.373
7.370
7.361
7.342
7.338
7.323
7.235
7.225
7.220
7.197
7.125
7.084
6.531
NH
3na
1.00
1.00
3.09
3.08
3.25
1.18
1.00
11.5 11.0 10.5 10.0 9.5 9.0 8.5 8.0 7.5 7.0 6.5 6.0 5.5 5.0 4.5 4.0 3.5 3.0 2.5 2.0 1.5 1.0 0.5 0.0
f1 (ppm)

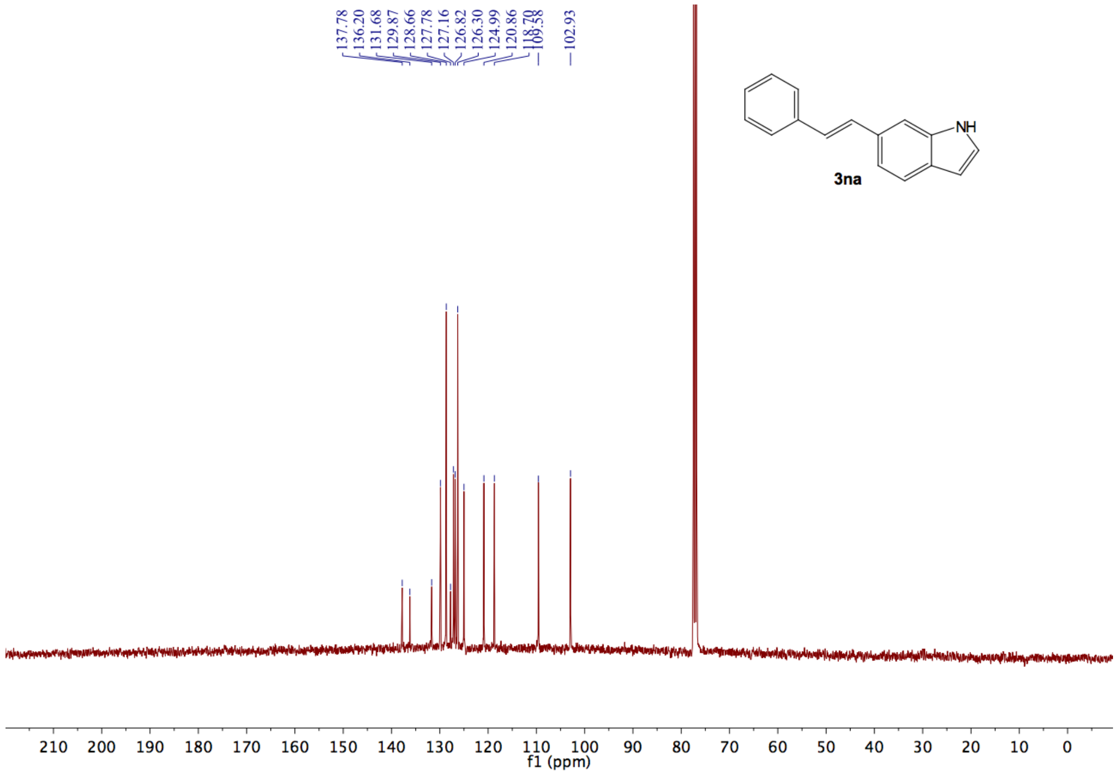
137.78
136.20
131.68
129.87
128.66
127.78
127.16
126.82
126.30
124.99
120.86
118.70
109.58
102.93
NH
3na
210 200 190 180 170 160 150 140 130 120 110 100 90 80 70 60 50 40 30 20 10 0
f1 (ppm)

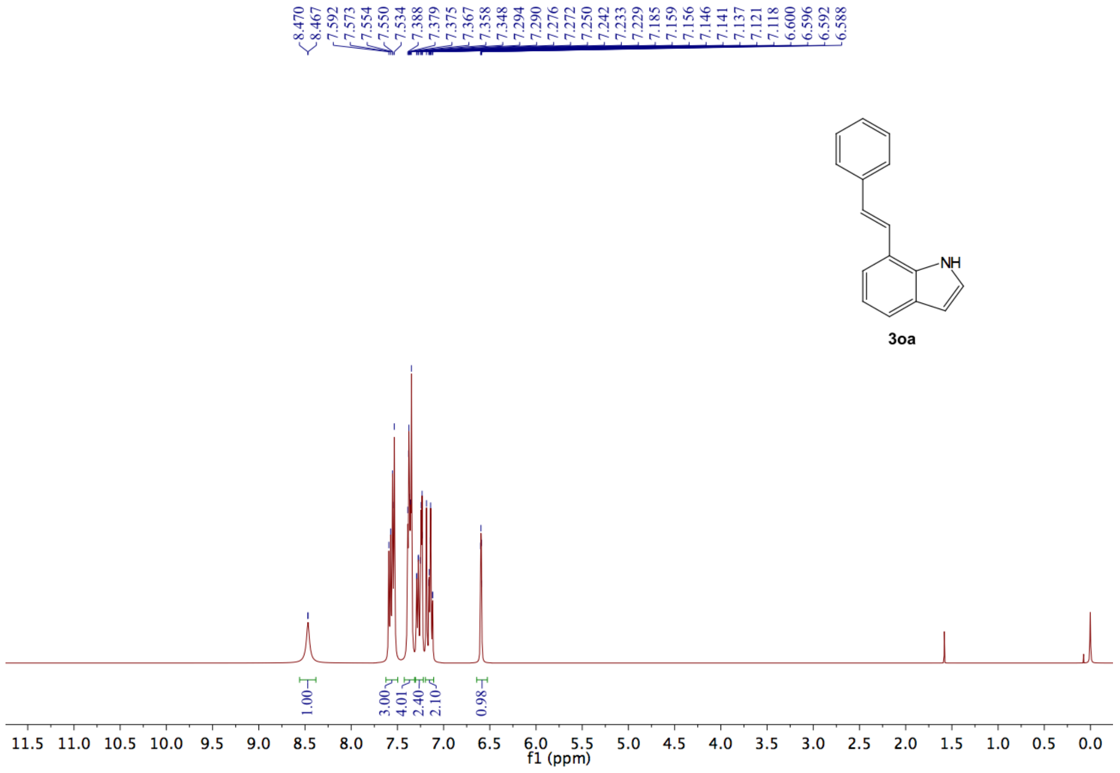
8.470
8.467
7.592
7.573
7.554
7.550
7.534
7.388
7.379
7.375
7.367
7.358
7.348
7.294
7.290
7.276
7.272
7.250
7.242
7.233
7.229
7.185
7.159
7.156
7.146
7.141
7.137
7.121
7.118
6.600
6.596
6.592
6.588
NH
1.00
3.00
4.01
2.40
2.10
0.98
11.5 11.0 10.5 10.0 9.5 9.0 8.5 8.0 7.5 7.0 6.5 6.0 5.5 5.0 4.5 4.0 3.5 3.0 2.5 2.0 1.5 1.0 0.5 0.0
f1 (ppm)

3oa

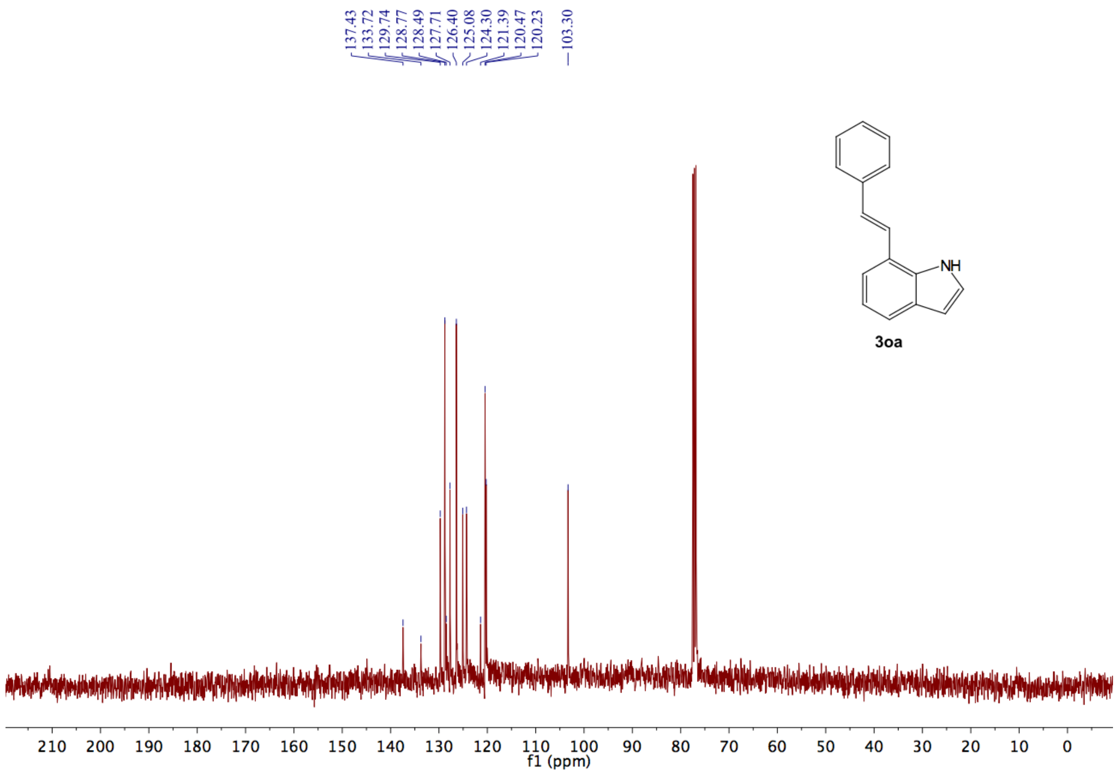

5. References

1. Zhu, X. Y.; Liu, J.; Chen, T.; Su, W. K. *Appl. Organomet. Chem,* **2012,** *26,* 147-147.

2. Xu, H. J.; Zhao, Y. Q.; Zhou, X. F. *J. Org. Chem,* **2011,** *76,* 8036-8041.

3. Yang, X. X.; Jin, X. Q.; Wang, C. Y. *Adv. Synth. Catal,* **2016,** *358*, 2436-2442.

4. Kanagaraj, K.; Pitchumani, K. *Chem-Eur. J,* **2013,** *19,* 14425-14431.

5. Kim. B. M.; Park. J. K. *Bull. Korean. Chem.Soc,* **1999,** *20*, 744-746.

6. Nakao, Y.; Imanaka, H.; Sahoo, A. K.; Yada, A.; Hiyama, T. *J. Am. Chem. Soc,* **2005,** *127,* 6952-6953.

7. Hutchison, A. J.; Williams, M.; Dejesus, R.; Yokoyama, R.; Oei, H. H.; Ghai, G. R.; Webb, R. L.; Zoganas, H. C.; Stone, G. A.; Jarvis, M. F. *J. Med. Chem,* **1990,** *33*, 1919-1924.

8. Yang, J. S.; Liau, K. L.; Li, C. Y.; Chen, M. Y, *J. Am. Chem. Soc,* **2007,** *129*, 13183-13192.

9. Molander, G. A.; Bernardi, C. R. *J. Org. Chem,* **2002,** *67*, 8424-8429.
